# Effectiveness of Exercise Treatments with or without Adjuncts for Common Lower Limb Tendinopathies: A Living Systematic Review and Network Meta-analysis

**DOI:** 10.1186/s40798-023-00616-1

**Published:** 2023-08-09

**Authors:** Dimitris Challoumas, Gearoid Crosbie, Seth O’Neill, Carles Pedret, Neal L. Millar

**Affiliations:** 1 School of Infection and Immunity, College of Medicine, Veterinary and Life Sciences, University of Glasgow, 120 University Avenue, Glasgow, G12 8TA UK; 2 Department of Physiotherapy, School of Allied Health Professionals, University of Leicester, Leicester, UK; 3 Sports Medicine and Imaging Department, Clinica Diagonal,, C/San Mateu, Esplugues de Llobregat, Spain

**Keywords:** Tendinopathy, Exercise, Achilles, Patellar, Greater trochanteric pain syndrome

## Abstract

**Introduction:**

Exercise therapy is usually prescribed as first-line treatment for lower limb tendinopathies. The multitude of exercise- and non-exercise-based management options can be overwhelming for the treating sports professional and patient alike. We chose to investigate the comparative effectiveness of exercise therapy with or without adjuncts for managing the commonest lower limb tendinopathies.

**Methods:**

Through an extensive systematic literature search using multiple databases, we aimed to identify eligible randomised controlled trials (RCTs) on Achilles tendinopathy, patellar tendinopathy or greater trochanteric pain syndrome (GTPS) that included at least one exercise intervention in their treatment arms. Our primary outcomes were patient-reported pain and function (Victorian Institute of Sport Assessment; VISA). Follow-up was defined as short-term (≤ 12 weeks), mid-term (> 12 weeks to < 12 months) and long-term (≥ 12 months). The risk of bias and strength of evidence were assessed with the Cochrane Collaboration and GRADE-NMA tools, respectively. Analyses were performed separately for each one of the three tendinopathies.

**Results:**

A total of 68 RCTs were included in the systematic review. All pairwise comparisons that demonstrated statistically and clinically significant differences between interventions were based on low or very low strength of evidence. Based on evidence of moderate strength, the addition of extracorporeal shockwave therapy to eccentric exercise in patellar tendinopathy was associated with no short-term benefit in pain or VISA-P. From the network meta-analyses, promising interventions such as slow resistance exercise and therapies administered alongside eccentric exercise, such as topical glyceryl trinitrate for patellar tendinopathy and high-volume injection with corticosteroid for Achilles tendinopathy were based on low/very low strength of evidence.

**Conclusion:**

In this network meta-analysis, we found no convincing evidence that any adjuncts administered on their own or alongside exercise are more effective than exercise alone. Therefore, we recommend that exercise monotherapy continues to be offered as first-line treatment for patients with Achilles and patellar tendinopathies and GTPS for at least 3 months before an adjunct is considered. We provide treatment recommendations for each tendinopathy.

PROSPERO registration number CRD42021289534.

**Supplementary Information:**

The online version contains supplementary material available at 10.1186/s40798-023-00616-1.

## Key Points

- Several treatment modalities are used early on in managing lower limb tendinopathies without sufficient evidence to support their use.
- Extracorporeal shockwave therapy does not appear to add any pain-relieving or functional benefits when added to eccentric exercise in patellar tendinopathy.
- Exercise interventions other than eccentric exercise and adjuncts to eccentric exercise appear promising but need further high-quality evidence.

## Introduction

Tendinopathy is a pathological state classically characterised by persistent pain and functional impairment due to damaged tendons. The tendon damage is secondary to abnormalities in its microstructure, composition and cellularity, usually as a result of repetitive mechanical overload [1, 2]. The primary aetiology of tendinopathy is linked to an impaired healing response secondary to repetitive tendon stress and overload [1–3]. Equally, it has been demonstrated that physical activity alone does not account for the extent of histopathological changes, suggesting that other factors are at the root cause of its aetiology [4]. Psychosocial factors are increasingly recognised to contribute to tendinopathy, and a biopsychosocial approach has been recommended for its treatment, which addresses the biological, psychological and social contributions to chronic pain and disability [5]. The tendons most commonly affected in the lower limb are the Achilles, patellar and gluteal (greater trochanteric pain syndrome; GTPS).

Whilst exercise has been implicated in inducing this condition, over the past 3 decades, strong evidence has demonstrated that rehabilitation with progressive loading improves pain and functional outcomes in tendinopathy [6]. The adaptive potential of tendons secondary to exercise has been demonstrated in several studies and there are multiple variables that may play a role and need to be considered for exercise interventions, such as intensity, load, frequency, muscle contraction type and repetitions [7–9]. Recent evidence suggests that loading magnitude and muscle contraction intensity may be more important than muscle contraction type [9].

The management of lower limb tendinopathies is complex. The need for long and often successive treatment modalities poses significant health and economic burden, which in conjunction with the often-debilitating effects of these conditions, make identification of the most effective treatments imperative [10, 11]. A significant proportion of patients do not regain full function after treatment, and chronic symptoms persist in approximately one-quarter of patients [12]. Over recent years, numerous therapeutic modalities have been assessed for treating lower limb tendinopathies, usually in conjunction with exercise therapy. These include, but are not limited to, extracorporeal shockwave therapy (ESWT), orthotics, ultrasound therapy (UST), laser therapy (LT), topical glyceryl trinitrate (GTN) therapy, corticosteroid (CS), platelet-rich plasma (PRP) and high-volume (HVI) injections. Surgery is reserved for treatment-resistant tendinopathy; however, the efficacy of surgical treatment is far from certain [12]. With the plethora of available interventions applied alone or in combination regimes, there is an increasing need for high-quality comparative studies and an overall assessment of the combined evidence that will inform management decisions and protocols.

The aim of the present study was to expand on our previous systematic review of patellar tendinopathy [13] and provide a more comprehensive living systematic review and network meta-analysis comparing the effectiveness of exercise interventions with or without adjunct treatments to other treatments or no treatment in patients with the commonest lower limb tendinopathies (Achilles, patellar tendinopathy and GTPS) with regard to patient-reported pain and function.

## Methods

This living systematic review and network meta-analysis (NMA) was prospectively registered on PROSPERO (CRD42021289534), conducted and authored as per PRISMA-NMA” and “PERSiST” guidance [14, 15]. This review was administered at the Institute of Infection, Immunity and Inflammation, University of Glasgow, Scotland, and we plan to update the NMA annually for a minimum of 5 years. We intend to screen the literature annually to identify new eligible data and re-perform analyses where necessary. When new data become available, we will update the analysis and present the updated findings on the website of Glasgow University (https://www.gla.ac.uk/researchinstitutes/iii/staff/nealmillar/). A plain-language summary for patients and clinicians dealing with lower limb tendinopathies will also be provided.

### Eligibility Criteria

#### Types of Studies

Randomised controlled trials (RCTs) of any type that investigated patellar and Achilles tendinopathies and GTPS were eligible for inclusion. Only studies published in English were screened for inclusion.

#### Types of Participants

Studies of patients with mid-portion Achilles tendinopathy, patellar tendinopathy or GTPS were eligible for inclusion. A clinical diagnosis of Achilles tendinopathy, patellar tendinopathy or GTPS made by a medical professional was required for inclusion with or without radiographic confirmation. Diagnostic criteria for each type of tendinopathy were not controlled for. Duration of symptoms or level of physical activity were not exclusion criteria for the trials. The present study excluded trials with (1) patients with insertional Achilles tendinopathy, (2) trials with participants < 18 years old, (3) partial/complete tendon rupture, (4) previous tendon surgery, and (5) animal or in vitro studies.

#### Types of Interventions

Studies assessing the effectiveness of any form and duration of exercise therapy with or without adjunct treatments were included. Both supervised and non-supervised exercise programmes were eligible. Any type of intervention that was administered alongside exercise therapy was considered an adjunct treatment.

#### Types of Comparators

Any type of treatment (exercise or non-exercise-based), placebo/sham treatment or no treatment that was compared to an exercise therapy with or without adjunct treatments was considered as a comparator.

#### Types of Outcome Measures

The primary outcomes were patient-reported pain and function, measured by the visual analogue scale (VAS) or equivalent (0–10 or 0–100), and the Victorian Institute of Sport Assessment (VISA) scale (0–100), respectively. The VISA questionnaire has been developed into distinct questionnaires to assess pain and functional outcomes in Achilles (VISA-A) and patellar (VISA-P) tendinopathies and GTPS (VISA-G) [16–18]. For the purpose of analysis and pooling of results, outcome measures were divided into three distinct intervals, short-term (≤ 12 weeks), mid-term (> 12 weeks to  < 12 months) and long-term (≥ 12 months). No secondary outcomes were assessed. Where studies reported results at more than one time point within our pre-specified intervals, those closest to the longer end of the interval were used.

When trials used different types of patient-reported pain, the following hierarchy was used: (a) pain at rest, (b) pain with (any) activity, (c) pain during sports, (d) pain during the day, (e) pain at night, (f) current pain.

### Literature Search

Search strategies were developed for each lower limb tendinopathy in “all fields” with the following Boolean operators: (a) ‘(patellar tendin* OR jumper’s knee), (b) ‘(Achilles tendin*), (c) ‘(gluteal tendin* OR greater trochanteric pain syndrome OR GTPS) AND (treatment OR management OR therapy OR intervention OR shockwave OR exercise OR physiotherapy OR loading OR eccentric OR concentric OR platelet-rich plasma OR PRP OR glyceryl trinitrate OR GTN OR *steroid OR glucocorticoid OR injection OR laser OR acupuncture OR orthotics)’.

**Fig. 1:**
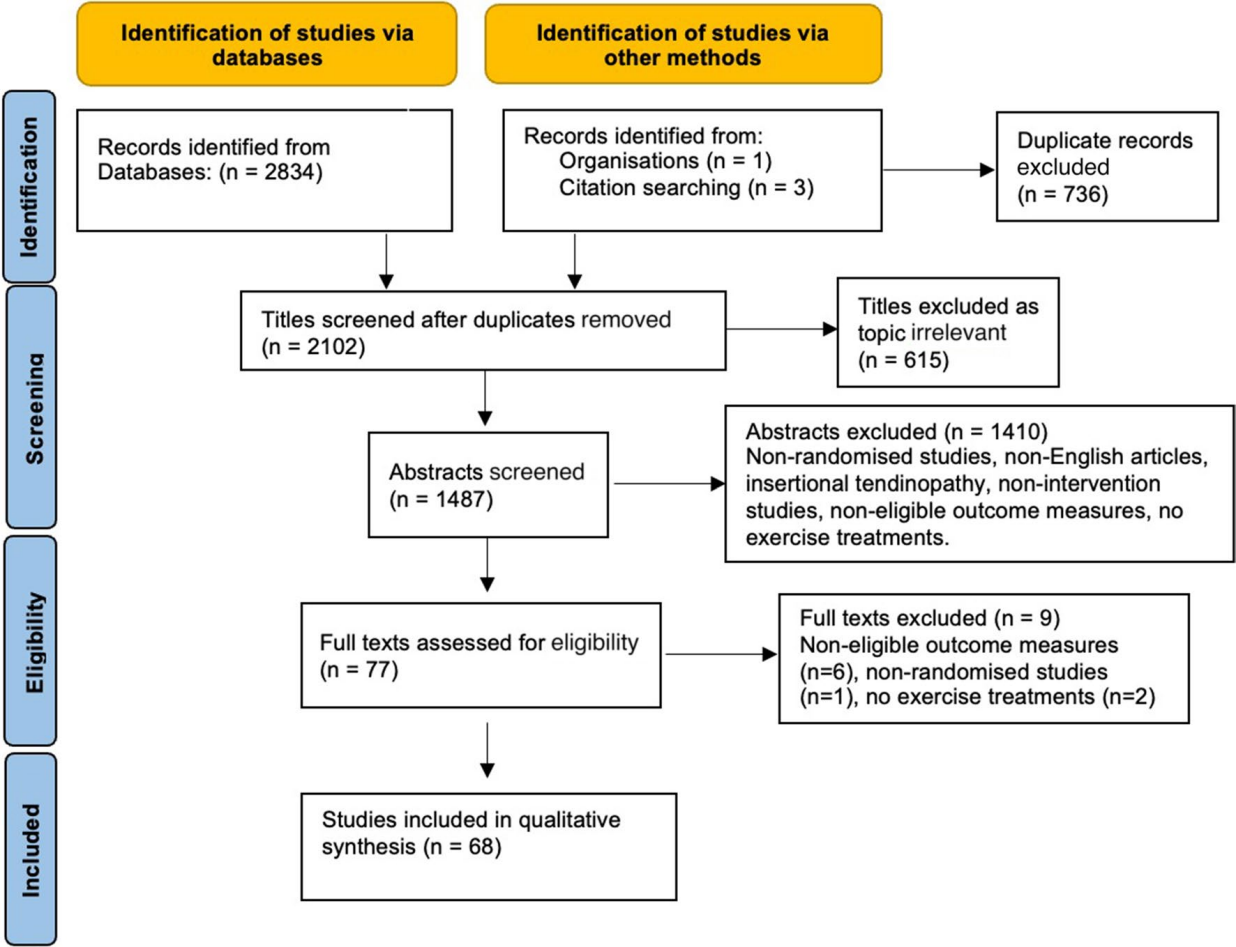
PRISMA flow diagram summarising the article selection process

The following databases were screened for published and unpublished trials from inception to 15/03/22 by a single author: Medline, Embase, PubMed, Cochrane Central, Scopus, CINAHL SPORTDiscus, OpenGrey.eu and WorldCat.org. For unpublished or ongoing studies, we searched the WHO International Clinical Trials Registry Platform (http://apps.who.int/trialsearch/) Clinical Trials.gov, The European Union Clinical Trials Register and the ISRCTN registry. All eligible studies' reference and citation lists were screened for further eligible trials. The PRISMA flow-chart is illustrated in Fig. 1.

### Data Extraction

Patient characteristics, duration of symptoms, nature of the therapeutic interventions, exercise programme description, outcome measures, and follow-up time points were extracted from individual trials and recorded in Microsoft Word version 16.43 (Microsoft Corporation) by two authors (GC and DC) in a previously constructed data extraction table. Additional file 1 was utilised where available for descriptive statistics. For missing data, attempts were made to contact the corresponding authors of studies published in the last 5 years, and if these were unsuccessful, the papers were excluded.

### Data Handling: Synthesis of Results

Comparisons of interventions reported by two or more studies at similar follow-up time points were pooled quantitatively by pairwise meta-analyses in the absence of significant clinical heterogeneity (similar populations, follow-up time points and interventions). Only studies of patients with the same type of tendinopathy were pooled. Raw mean differences (MD) with their accompanying 95% confidence interval (CI) were calculated and used in the tests as the tools used across studies were the same. Finally, a network meta-analysis was conducted for both outcome measures (pain VAS and VISA) at each follow-up period where adequate data existed.

For the present article, “Achilles tendinopathy” refers to non-insertional Achilles tendinopathy only.

### Risk of Bias and Strength of Evidence Assessment

The Cochrane Internal Validity Tool was used to assess the risk of bias for each RCT [19]. Studies were assessed by two authors separately (GC and DC), and disagreements were resolved with the involvement of the senior author (NLM). The overall risk of bias for each RCT was labelled as low or high based on how likely the assessor thought that the presence of high risk of bias in the individual domains was to affect the true outcomes of the assessed interventions. We avoided using pre-specified criteria (e.g. overall “high risk” if specific domains of the tool or a certain number of them were of high risk of bias for each study) as we believe that the assessor’s judgement was more fair and accurate for determining the overall risk of bias.

The Grading of Recommendations Assessment, Development and Evaluation for network meta-analysis (GRADE-NMA) was used to appraise the strength (certainty) of evidence [20]. For pairwise meta-analyses, the strength of evidence was assessed based on four domains: overall risk of bias, imprecision, inconsistency and other confounding factors (including publication bias). The result of each comparison of interventions arising from the pooling of similar studies was assigned one of high, moderate, low or very low strength of evidence. This process was completed independently by two authors (GC and DC) for each outcome measure, and disagreements were resolved by the involvement of the senior author (NLM). For network meta-analyses, the strength of both the direct and the indirect evidence was taken into account as per GRADE-NMA, along with intransitivity (clinical heterogeneity of studies participating in direct versus those participating in indirect evidence) and similarly to pairwise meta-analyses, the results were assigned one of the aforementioned four strengths of evidence. Recommendations for clinical practice were given only based on results of high or moderate strength of evidence. An intervention was thought to be more effective than another intervention when its superiority was based on both statistical and clinical significance.

For network meta-analyses, the strength of evidence of the direct estimate was rated first using the overall risk of bias, inconsistency (statistical heterogeneity), indirectness (clinical heterogeneity) and publication bias (GRADE-NMA tool). Subsequently, the indirect estimate was rated using the lowest of the ratings of the two direct comparisons forming the most dominant first-order loops and intransitivity (differences in study characteristics of studies used in indirect comparisons). Finally, the network estimate was rated using the highest certainty of evidence between direct and indirect estimates, the incoherence (difference between direct and indirect comparisons—assessed using the “node splitting” approach) and imprecision.

### Statistical Analysis

The Review Manager V.5 (RevMan) software was used to calculate the pooled mean difference and generate forest plots for pairwise meta-analyses and their accompanying heterogeneity tests (Chi^2^ and I^2^). STATA 16.1 with Ian White’s extension (multivariate random-effects meta-regression) was used for network meta-analyses (frequentist approach) [21]. When exact numerical mean, mean difference or standard deviation (SD) values were not recorded in the individual papers, an estimated value was extrapolated from available graphs. When the results were recorded as mean difference and interquartile range (IQRs), the SD value was derived as IQR divided by 1.35. Where median values were reported, the mean was assumed to be the same. The RevMan software was used to generate SD values when only confidence intervals were recorded in trials. In studies in which only mean values were presented without SDs, the prognostic method described by Ma et al. [22] was used to generate an SD by calculating the mean of all the other SDs in that comparison.

Pooled SDs were calculated with the following formula:

$${\text{SD}}_{{{\text{pooled}}}} = \surd \left[ {{\text{SD}}_{1}^{2} \left( {n_{1} - 1} \right)} \right] + \, \left[ {{\text{SD}}_{2}^{2} \left( {n_{2} - 1} \right)} \right] \, + \cdots + \, \left[ {{\text{SD}}_{k}^{2} \left( {n_{k} - 1} \right)} \right]/\left( {n_{1} + \, n_{2} + \cdots + \, n_{k} {-} \, k} \right),$$

where SD = standard deviation, *n* = sample size, *k* = number of samples.

The following formula was used for the sample size calculation as part of GRADE’s assessment for imprecision:

$$N = \frac{{2\left( {a + b} \right)^{2} {\text{SD}}^{2} }}{{x^{2} }}$$

where *N* = the sample size required in each of the groups (optimal information size), *x* = minimal clinically relevant difference (MCRD); defined as 1.5 points for pain VAS and 13 points for VISA, SD^2^ = population variance (calculated using pooled SD from included treatment groups), *a* = 1.96 (for 5% type I error), *b* = 0.842 (for 80% power).

The optimal information size (minimum number of overall patients combined in each meta-analysis for sufficient “precision” in the GRADE assessment) with the use of the above formula was calculated as *N* = 34 patients for pain and *N* = 20 for VISA.

Potential publication bias was not assessed as no pairwise meta-analyses included more than 10 studies. Expecting wide-range variability in studies’ settings, a random-effects model was employed in all meta-analyses. Where heterogeneity was found to be high (*I*^2^ = 50–75%), the strength of evidence was downgraded by one level; where it was found to be substantial (*I*^2^ > 75%), it was downgraded by two levels.

Subgroup and sensitivity analyses were not performed as all pairwise meta-analyses had a maximum of only three RCTs.

### Protocol Deviations

There were no deviations from the pre-defined protocol.

### Definition of Loading Types

*Isotonic—eccentric* exercise types involving muscle contractions which result in movement of a limb and lengthening of the muscle unit.

*Isotonic—concentric* exercise types involving muscle contractions which result in movement of a limb and shortening of the muscle unit.

*Isometric* exercise types involving muscle contractions which result in no movement of a limb. The muscle unit may shorten marginally as the tendon elongates whilst under a contraction.

*Slow resistance* slow, isotonic exercises utilising both eccentric and concentric phases. Depending on the load used relative to repetition maximum (maximum load an individual can use for a defined number of repetitions), this can be “heavy” or “moderate” slow resistance.

## Results

A total of 2834 studies were initially identified from the searches. An additional four studies were identified from other sources. After removing duplicate results and non-eligible study types, the titles (*n* = 2102), abstracts (*n* = 1487) and full texts (*n* = 77) of the remaining studies were screened for inclusion by two authors independently. A total of 68 studies [23–90] were found to be eligible and were included in the systematic review, 31 assessing interventions in Achilles tendinopathy (*n* = 1792 patients, mean age 46 years), 31 in patellar tendinopathy (*n* = 1109 patients, mean age 28 years) and 6 in GTPS (*n* = 907 patients, mean age 55 years). The majority of outcome measures were assessed at short- and mid-term follow-up. Five (5) studies assessed immediate post-intervention outcomes only [31, 44, 63, 64, 82]. Patient and trial characteristics (Additional file 1: Tables S1–S3), risk of bias assessment results (Additional file 1: Tables S4–S6), strength of evidence assessment (Additional file 1: Table S7) and the tabulated results of the compared interventions (Additional file 1: Tables S8–S10) can be found in the supplementary material. The most common type of pain reported was “with single decline squat” in patellar tendinopathy (7 studies), “at rest” in Achilles tendinopathy (11 studies), and “over the last week” (5 studies) for GTPS.

### Pairwise Meta-analyses

**Fig. 2:**
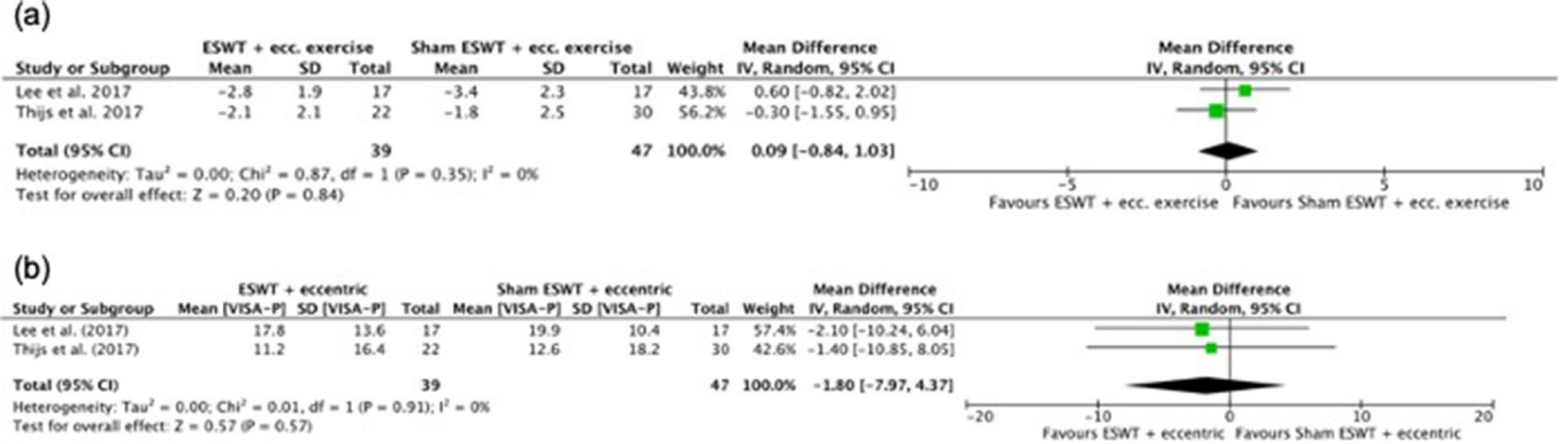
Meta-analysis results and forest plots of eccentric exercise plus placebo (control) versus eccentric exercise plus ESWT (intervention) for **a** short-term pain VAS in patellar tendinopathy and **b** short-term VISA-P in patellar tendinopathy

The pairwise comparisons of interventions are presented below for each tendinopathy type. The forest plots of results based on moderate or high strength of evidence are shown in Fig. 2, whilst those of low or very low strength of evidence can be found in the Additional file 1: Figures S1–S4. For each result, the accompanying parentheses show the MD of the pairwise comparison with its confidence interval and heterogeneity test and the number of pooled studies.

### Achilles Tendinopathy

#### Eccentric Exercise Plus PRP Injection Versus Eccentric Exercise Plus Placebo

*Short-term and Mid-term follow-up* Based on evidence of low strength, there was no difference in VISA-A between the eccentric exercise *plus* PRP injection group and the eccentric exercise *plus* placebo injection groups at short-term follow-up [MD 1.52, 95%CI (− 2.49, 5.53), *I*^2^ = 5%, *P* = 0.46, 3 studies] (Additional file 1: Figure S1a). At mid-term follow-up, there was a difference in VISA-A in favour of the eccentric *plus* PRP injection group when compared to the eccentric group *plus* placebo injection based on low strength of evidence, though not likely to be clinically significant [MD 5.05, 95% CI (1.45, 8.64), *I*^2^ = 0%, *P* = 0.006, 3 studies] (Additional file 1: Figure S1b).

#### Eccentric Exercise *Plus* Low-Level Laser Therapy Versus Eccentric Exercise *Plus* Placebo

*Short-term follow-up* For VISA-A, we found very low strength evidence for a difference in favour of the eccentric exercise *plus* low-level LT when compared to eccentric exercise *plus* placebo at short-term follow-up, which is likely not clinically significant [MD 6.29, 95% CI (1.72, 10.85), *I*^2^ = 85%, *P* = 0.007, very low strength, 2 studies] (Additional file 1: Figure S2).

### Greater Trochanteric Pain Syndrome

#### Corticosteroid Injection Versus Exercise Therapy

*Short-term and long-term follow-up* We found low strength evidence for a difference in pain VAS scores favouring CS injection when compared to exercise therapy (mixed types) in patients with GTPS at short-term follow-up; this is likely to be clinically significant [MD 1.57, 95% CI (1.1. 2.1), *I*^2^ = 94%, *P* = 0.01, 2 studies] (Additional file 1: Figure S3a). There was no difference in reported pain VAS scores between the groups at long-term follow-up based on evidence of low strength [MD − 0.7, 95% CI (− 0.6, 0.45), *I*^2^ = 97%, *P* = 0.78, 2 studies] (Additional file 1: Figure S3b).

### Patellar Tendinopathy

#### Eccentric Exercise *Plus* ESWT Versus Eccentric Exercise *Plus* Placebo

*Short-term* Based on moderate strength evidence, there was no difference in short-term pain VAS or VISA-P between eccentric exercise *plus* ESWT and eccentric exercise *plus* placebo ESWT [MD − 0.09, 95% CI (− 1, 0.84), *I*^2^ = 0%, *P* = 0.84, moderate strength, 2 studies; MD − 1.8, 95% CI (− 8, 4.4), *I*^2^ = 0%, *P* = 0.57, 2 studies, respectively] (Fig. 2).

#### Isotonic Exercise Versus Isometric Exercise

*Short-term follow-up* We found low strength evidence for no differences in pain VAS immediately post-intervention with isometric versus isotonic exercise in patients with patellar tendinopathy [MD 1.03, 95% CI (− 2.57, 0.5), *I*^2^ = 70%, *P* = 0.19, 3 studies] (Additional file 1: Figure S4).

#### Network Meta-analysis

**Fig. 3:**
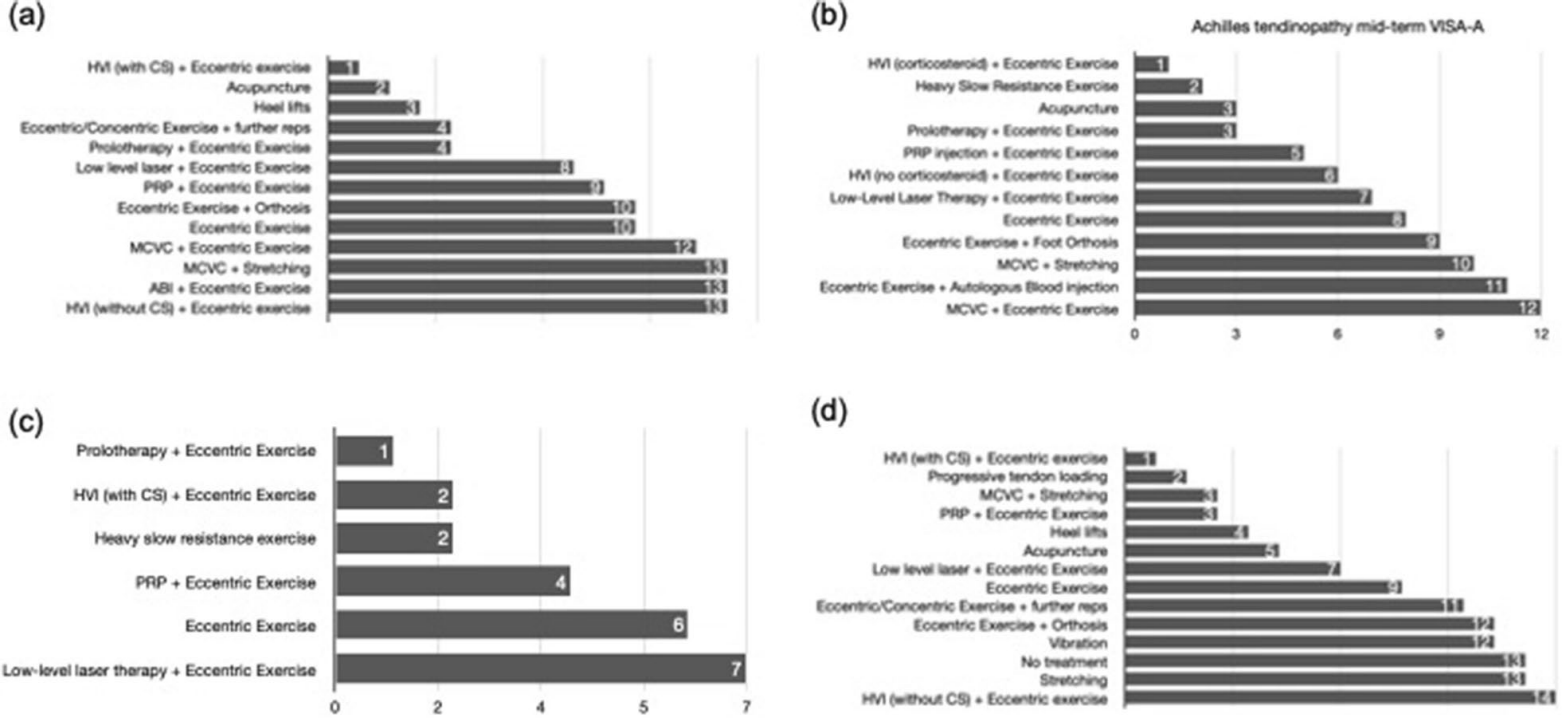
Median ranks of the effectiveness of interventions included in the network meta-analysis for **a** short-term VISA-A in Achilles tendinopathy. **b** mid-term VISA-A in Achilles tendinopathy, **c** for long-term VISA-A in Achilles tendinopathy and **d** short-term pain VAS in Achilles tendinopathy. *PRP* platelet-rich plasma, *HVI* high volume injection, *MVCS* mucopolysaccharides, type I collagen injection plus vitamin supplementation, *ABI* autologous blood injection, *CS* corticosteroid

The network maps, network forest plots and comparative treatment class effects deriving from all performed network meta-analyses can be found in the Additional file 1: Figures S5–S10. Figure 3 illustrates the median treatment ranks for each tendinopathy for pain VAS and VISA at each follow-up time intervals where data were available. A total of 17 and 19 interventions participated in the network meta-analyses for Achilles and patellar tendinopathy, respectively. Data were insufficient for network meta-analyses in GTPS.

### Achilles Tendinopathy

*Short-term follow-up* Eccentric exercise *plus* HVI (with CS) had the highest probability of being the most effective treatment for improving VISA-A and pain VAS scores (probability 56% and 45%, respectively). Passive stretching *plus* a dietary supplement containing mucopolysaccharides, type I collagen and vitamin C (MCVC) and eccentric exercise plus HVI (without CS) had the highest probability of being the least effective treatment for VISA-A (probability 22% and 46%, respectively) (Fig. 3a, b).

*Mid-term follow-up* Eccentric exercise *plus* HVI (with CS) had the highest probability (83%) of being the most effective treatment for improving VISA-A, whilst eccentric exercise *plus* MCVC dietary supplement had the highest probability (42%) of being the least effective treatment for improving VISA-A (Fig. 3c).

*Long-term follow-up* Eccentric exercise plus prolotherapy had the highest probability (55%) of being the most effective treatment for improving VISA-A scores, closely followed by eccentric exercise *plus* HVI (with CS) and heavy slow resistance exercise. Combination treatment with low-level LT *plus* eccentric exercise had the highest probability of being the least effective treatment (44%) (Fig. 3d).

### Patellar Tendinopathy

**Fig. 4:**
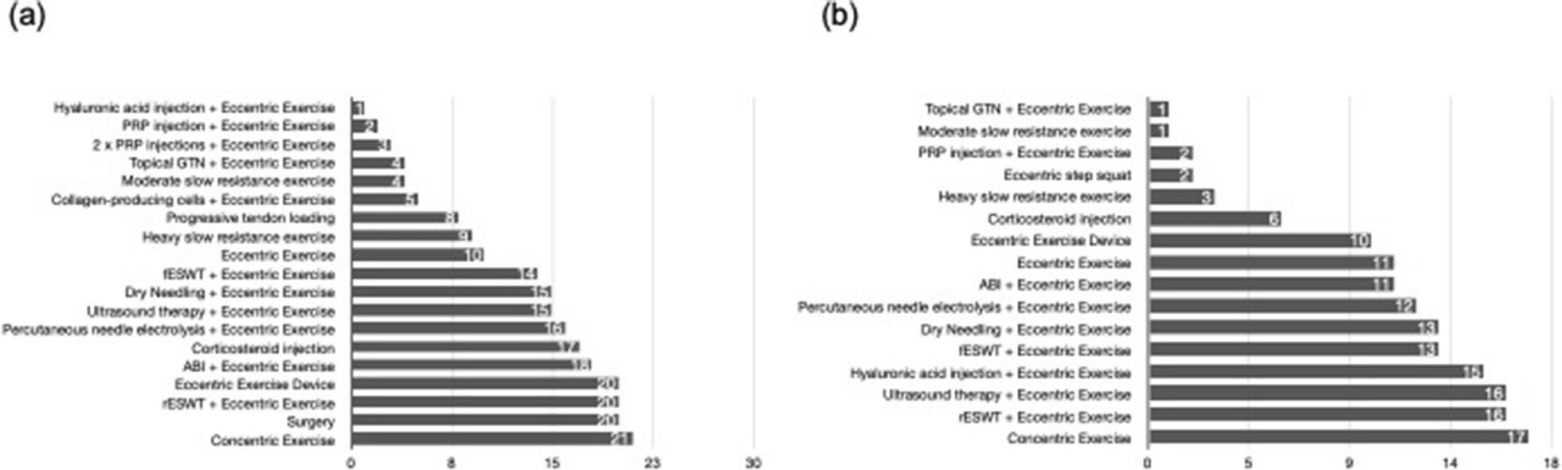
Median ranks of the effectiveness of interventions included in the network meta-analysis for **a** short-term VISA-P in patellar tendinopathy and **b** short-term pain VAS in patellar tendinopathy. *PRP* platelet-rich plasma, *GTN* glyceryl trinitrate, *rESWT* radial extracorporeal shock wave therapy, *fESWT* focal extracorporeal shock wave therapy

*Short-term follow-up* For VISA-P, combination therapy with eccentric exercise *plus* hyaluronic acid injections had the highest probability (75%) of being the most effective intervention, whilst concentric exercise had the highest probability (42%) of being the least effective intervention (Fig. 4a). For pain VAS, slow resistance exercise of moderate load had the highest probability (22%) of being the most effective treatment, closely followed by eccentric exercise *plus* topical GTN, whilst concentric exercise had the highest probability (48%) of being the least effective treatment (Fig. 4b).

*Mid-term and long-term follow-up* Data were insufficient for network meta-analyses in patellar tendinopathy at these follow-up time intervals.

#### Network Meta-analyses Strength of Evidence

For both Achilles and patellar tendinopathy, all results arising from network meta-analyses presented both in text and in figures are considered as of low/very low strength of evidence due to the high overall risk of bias of the included studies and intransitivity.

## Discussion

The mainstay of treatment for lower limb tendinopathies is non-operative. Exercise therapy for at least 3 months results in an improvement in function and pain for the majority of patients [3, 6, 91]. Amongst different exercise programmes, eccentric exercise has been thought to be the gold standard management for patellar and Achilles tendinopathy and is usually prescribed as first-line treatment. The effectiveness of eccentric loading for these two tendinopathies is embraced by the research community; the vast majority of non-exercise interventions being assessed were administered alongside eccentric exercise. As a result, conclusions on the effectiveness of these interventions as monotherapies cannot be made. They should always be prescribed as an adjunct to a continuous exercise programme, which should constitute the main focus of the patient’s management. The safety and good tolerability of eccentric exercise make it an even more attractive option. However, it relies heavily on patient compliance, especially where financial constraints at an individual or healthcare system level preclude long-term supervised programmes [81]. For GTPS, eccentric loading on its own is not recommended as the gluteal tendons have short excursion and eccentric exercises alone would be very challenging.

Our study includes all eligible evidence on the comparative effectiveness of exercise interventions for patellar tendinopathy, Achilles tendinopathy and GTPS with or without adjuncts. Our only result which can be used for strong practice recommendations is the lack of benefit on pain or VISA-P of ESWT added to eccentric exercise for patellar tendinopathy (moderate strength of evidence)—therefore, we do not recommend its use. The remaining results were of low or very low strength of evidence. In the following few paragraphs, we discuss promising interventions arising from our findings for each lower limb tendinopathy separately and also summarise the results of other similar systematic reviews and relevant evidence. Based on these, we provide our treatment recommendations for each tendinopathy.

### Achilles Tendinopathy

For Achilles tendinopathy, there were no results of comparisons of interventions that were based on moderate or high strength of evidence. Based on evidence of low strength from our pairwise meta-analyses, PRP injections added to eccentric exercise were associated with no short-term benefit and a small, non-clinically significant mid-term benefit in VISA-A. Similarly, low-level laser therapy was associated with a small clinically insignificant short-term benefit in VISA-A when added to eccentric exercise.

From the network meta-analyses, HVI with CS combined with eccentric exercise ranked first in the network meta-analysis for short-term pain VAS and VISA-A, and it also ranked first for mid-term VISA-A improvement. Long-term VISA-A increases were likely to be the highest in patients receiving prolotherapy and eccentric exercise; however, only a single study contributed data for this treatment group. Heavy slow resistance exercise as monotherapy also ranked very highly (second) for mid-term and long-term VISA-A improvements and the single RCT that compared it to eccentric exercise found no differences at 12 weeks or 1 year for pain or VISA-A [28]. In the included RCTs, HVI with CS alongside eccentric exercise consistently demonstrated positive results, whilst those of HVI without CS combined with eccentric exercise were conflicting [30, 81]. It is not impossible that the clinical benefits of HVI with CS may be related to the CS alone, as demonstrated by a recent RCT that found that, added to heavy slow resistance exercise, US-guided CS injection was superior than a placebo injection for VISA-A in the short-, mid- and long-term [92]. HVI was administered under US guidance in all studies, and finally, HVI with or without CS was not associated with any complications; however, the very small (theoretical) risk of tendon rupture associated with locally-administered CS should always be taken into account and communicated to patients [81]. Finally, the limited published evidence on the effectiveness of isometric loading for immediate post-intervention pain relief in Achilles tendinopathy does not support its use [82, 93].

In their similar systematic review and network meta-analysis on Achilles tendinopathy, van der Vlist et al. [94] found no convincing evidence for the superiority of any interventions over others based on the lack of adequate strength of evidence, which is in agreement with our results. As a conclusion, they recommend a calf-muscle exercise programme to be prescribed as first-line treatment as it is easy, widely available, safe and cheap [94]. Another network meta-analysis by Rhim et al. [95] concluded that the addition of HVI with CS and ESWT to eccentric exercise could potentially improve long-term outcomes which is partly in agreement with our recommendations.

We recommend the use of a progressive eccentric or heavy slow resistance exercise for 12 weeks as first-line treatment for Achilles tendinopathy. For resistant cases (no or minimal improvement at the end of the programme), a US-guided HVI with CS could be added to the continuing exercise programme if available.

### Patellar Tendinopathy

Pairwise meta-analyses found no interventions that were superior to others for patellar tendinopathy. For short-term pain relief, promising interventions from the network meta-analyses included slow resistance exercise of moderate load and eccentric exercise combined with topical GTN. For short-term VISA-P improvements, eccentric exercise combined with hyaluronic acid injections ranked the highest. Only single studies contributed data to the networks for each of these promising interventions; therefore, clinical practice recommendations for their use can only be weak at this point. No complications were reported with the use of hyaluronic acid injections in the included studies, whilst topical GTN may cause headaches [47, 96]. In the relevant included RCT, two hyaluronic acid injections were administered under US guidance 1 week apart, whilst three injections 1 week apart have also been used successfully for Achilles and patellar tendinopathy elsewhere [47, 97, 98]. Topical GTN was used as one patch (5 mg) daily for 12 weeks in the included RCT [71]. Heavy slow resistance exercise was as effective as eccentric exercise up to 6 months of follow-up in one of the included RCTs and in another RCT slow resistance exercise of moderate load (55% of one repetition maximum) was no less effective than that of heavy load (90% of one repetition maximum) [24, 50]. There was adequate evidence (moderate strength) for strong clinical practice recommendations to suggest that ESWT does not appear to add any benefits when used with eccentric exercise for either pain or VISA-P in patellar tendinopathy.

Isometric exercise has been suggested for immediate pain reduction in patellar tendinopathy as isotonic loading can be painful [63]. Three RCTs compared a bout of isometric versus isotonic muscle contractions for immediate post-intervention pain relief in young athletes and reported conflicting results [44, 63, 64]. Our pairwise meta-analysis found a difference of 1 VAS point favouring isometric loading, which however did not reach clinical or statistical significance (low strength of evidence). Mid- and long-term follow-up results for the effectiveness of isometric exercises on patellar tendinopathy do not exist from RCTs.

In a recent systematic review and network meta-analysis, Chen et al. [99] found no benefits of adding ESWT to eccentric exercise in their pairwise meta-analyses, which is in agreement with our findings. Their network meta-analysis found that dry needling and PRP injections were the highest-ranked interventions. However, their network methodology may have been flawed as some of the studies were not in the network loop (i.e. not all studies had common comparators). Finally, in a previous systematic review of RCTs, topical GTN was superior to placebo in the management of tendinopathy (all types combined); however, its side effects, especially headaches, should be considered and explained to patients [96].

We recommend the use of a progressive eccentric exercise or slow resistance exercise (of “moderate” or “heavy” load) for 12 weeks as first-line treatment for patellar tendinopathy. For resistant cases (no or minimal improvement by the end of the programme), a course of topical GTN or a course of 2–3 US-guided hyaluronic acid injections could be added to the exercise regime depending on availability, and the patient’s and physician’s preference. Where isotonic exercise is not tolerated due to pain, isometric exercise may be used in the initial stages of the treatment regime. ESWT is not recommended for patellar tendinopathy.

### Greater Trochanteric Pain Syndrome

Gluteal tendinopathy, which has been renamed as the more generic term “GTPS”, has not received as much attention in the literature as the other two lower limb tendinopathies. This is evident from our inability to perform a network meta-analysis due to insufficient data. Our pairwise meta-analysis results (based on very low strength of evidence) suggest that, for short-term pain relief, a CS injection may be superior to exercise therapy (mixed types). These benefits were likely to be clinically significant. The short-term benefits of CS did not last in the mid-term or long-term. In a RCT that included a CS injection, a ESWT and a home exercise treatment group found that the CS injection group had lower pain scores 1 month after treatment but the reverse was true 15 months after treatment [68]. Also, a significantly larger proportion of those treated with ESWT or exercise had their symptoms resolved or improved at 15 months compared to those treated with a CS injection. The most commonly reported complication with ESWT was skin irritation. In another RCT, combined with a home exercise programme, ESWT was significantly more effective than sham ESWT at 2 months for pain relief. Additionally, ESWT combined with exercise had significantly greater pain-relieving benefits at long-term follow-up compared with CS injection combined with exercise in another RCT [42]. No tendon ruptures or other major complications associated with ESWT or CS injections were observed in any of these studies. All included RCTs administered 3 weekly sessions of ESWT. Finally, isometric loading was found to be as effective as isotonic loading for pain and functional outcomes up to 12 weeks in a RCT [34].

In their systematic review, Barratt et al. [100] also reported the superiority of CS injections compared to exercise for short-term pain relief. Additionally, CS injections yielded more favourable short-term outcomes for pain compared to ESWT and usual care. Their general conclusion on the management of GTPS was that definitive recommendations could not be made due to the lack of high-quality evidence. In a recent network meta-analysis, Gazendam et al. [101] found no interventions superior to no treatment at 6 and 12 months. However, PRP injections and ESWT may provide short-term pain relief, and structured exercise could produce short-term improvement in functional outcomes [101]. Wang et al. [102] found that the existing evidence on the effectiveness of CS injections in GTPS is equivocal. A RCT that was not included in our systematic review as the participants did not perform exercise as part of their treatments found no benefits of CS injections compared to placebo injections [103]. Despite the possible short-term pain-relieving benefits of CS injections in GTPS, we do not recommend its use due to the lack of longer-term benefits and their possible side effects. Finally, education of patients with GTPS may be at least as important as the exercise programme itself and stretching of the gluteal tendons should be avoided as compressive loading may be exacerbating tendinopathic pain [34, 104].

We recommend the use of a progressive isotonic exercise programme (utilising both eccentric and concentric phases) for 12 weeks as first-line treatment for GTPS. Where isotonic loading is not tolerated due to pain, an isometric programme could be prescribed instead, at least in the initial stages of treatment. In patients with restricted hip movements due to concomitant hip osteoarthritis, an isometric programme could be used for the whole duration of the treatment regime. Combined isotonic/isometric programmes could also be used as first-line treatment. For resistant cases (no or minimal improvement by the end of the programme), a ESWT programme (3 weekly sessions) could be added to exercise if available.

Table 1 summarises our treatment recommendations for each one of the three tendinopathies. For all three tendinopathies, patient education should constitute an integral part of management, and it should include explanation of principles of management and gradual load increases, advice on avoiding positions and activities that exacerbate symptoms and as much supervision as possible to maximise compliance and ensure the exercises are performed correctly.

**Table 1:** A summary of our management recommendations for Achilles and patellar tendinopathies and greater trochanteric pain syndrome

Tendinopathy	Recommended first-line treatment	Resistant cases
Achilles tendinopathy	Isotonic exercise programme (eccentric or heavy slow resistance)	Add high-volume injection with corticosteroid
Patellar tendinopathy	Isotonic exercise programme (eccentric or moderate/heavy slow resistance) OR Isometric exercise programme if isotonic exercise not tolerated	Add topical glyceryl trinitrate OR Add hyaluronic acid injections
Greater trochanteric pain syndrome	Isotonic exercise programme (concentric and eccentric) OR Isometric exercise programme if isotonic exercise not tolerated	Add extracorporeal shockwave therapyRemove programme

“Resistant cases” are those with minimal or no improvement of symptoms by the end of the exercise programme at 12 weeks. The recommended adjuncts for resistant cases should be added to a continuing exercise programme and not used as monotherapies

### Study Limitations

The most significant limitations of our study arise from the nature and inadequacy of the included evidence rather than methodological flaws at a meta-analysis level. We included all eligible RCTs and performed thorough assessment of the risk of bias and strength of evidence with pooling of results at pre-specified follow-up time points. However, the majority of results were based on low or very low strength of evidence. Placebo interventions were considered the same as no treatment when in reality, placebo could have its independent effects on tendinopathy. Many interventions were not connected to the network and therefore, could not be included in the network meta-analyses. For GTPS, the exercise treatment group was mixed including more than one type of loading. Finally, patient characteristics were not considered as subgroups would further decrease the strength of evidence; in reality, patients of different age groups, activity levels and duration of symptoms may respond differently to interventions.

## Conclusion

There appears to be no convincing evidence currently to suggest that any adjuncts administered alone or alongside exercise are superior to exercise monotherapy for the treatment of Achilles tendinopathy, patellar tendinopathy or GTPS. Therefore, we recommend that exercise monotherapy continues to be used as a first-line treatment for these tendinopathies, and it will be effective for most patients. For resistant cases, we provide recommendations for addition of adjuncts/alternative loading programmes based on our results and the rest of the published evidence; the evidence for these is of low or very low strength; therefore, these recommendations may change in the future if evidence of higher strength suggests the superiority of other treatments, further research should focus on promising interventions with inadequate strength of evidence and especially on the comparison of different loading types used as monotherapies to inform clinical practice with more certainty.

### Supplementary Information

**Additional file 1**. Supplementary meta analysis figures, network maps and tables.

## Data Availability

Data are available from DC upon request.
