# Supplementary material for: Effectiveness of Exercise Treatments with or without Adjuncts for Common Lower Limb Tendinopathies: A Living Systematic Review and Network Meta-analysis
Source: Sports Med Open. 2023 Aug 9;9:71. doi: 10.1186/s40798-023-00616-1 (PMC10409676; doi:10.1186/s40798-023-00616-1)
Supplement: Supplementary file 1 — Additional file 1. Supplementary meta analysis figures, network maps and tables. [file 40798_2023_616_MOESM1_ESM.docx]

**Supplementary material**

**Supplementary Figures
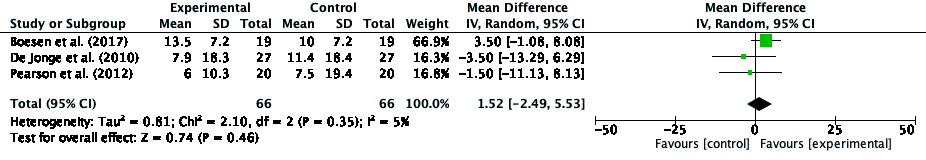
**

**Suppl. Figure 1a. Meta-analysis results and forest plot of eccentric exercise *plus* platelet-rich plasma injection (intervention) vs eccentric exercise *plus* sham injection (control) for short-term VISA-A in Achilles tendinopathy.**

**
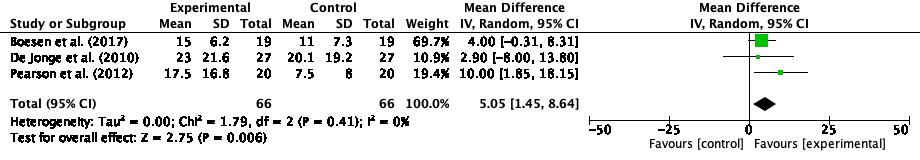
**

**Suppl. Figure S1b. Meta-analysis results and forest plot of eccentric exercise *plus* platelet-rich plasma injection (intervention) vs eccentric exercise *plus* placebo (control) for mid-term VISA-A in Achilles tendinopathy**

**
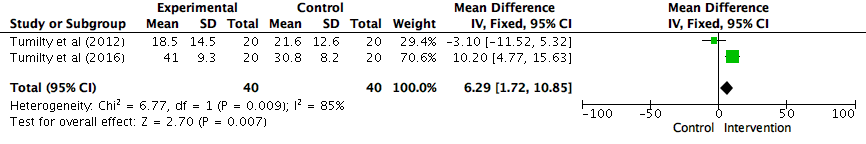
**

**Suppl. Figure S2. Meta-analysis results and forest plot of eccentric exercise *plus* low-level laser treatment (intervention) vs eccentric exercise *plus* placebo (control) for short-term VISA-A in Achilles tendinopathy**

**
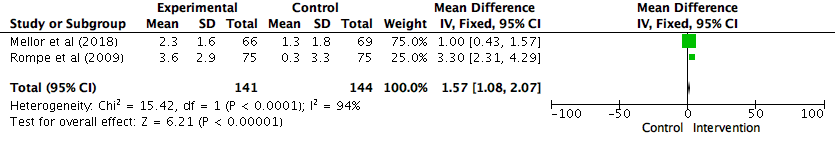
**

**Suppl. Figure S3a. Meta-analysis results and forest plot of exercise therapy (control) vs corticosteroid injection (intervention) for pain VAS at short-term follow up in GTPS**


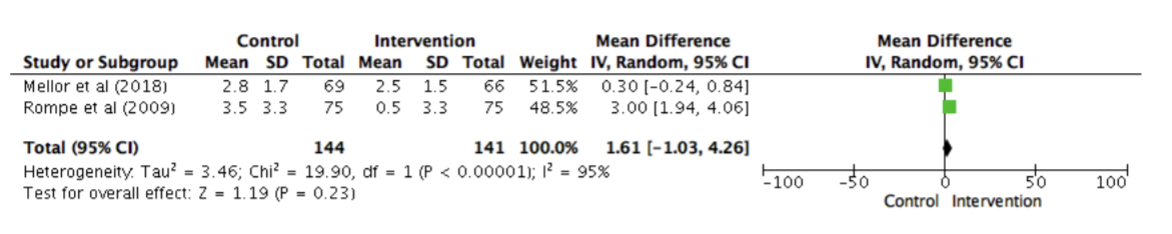


**Suppl. Figure S3b. Meta-analysis results and forest plot of exercise therapy (control) vs corticosteroid injection (intervention) for pain VAS at long-term follow up in GTPS**

**
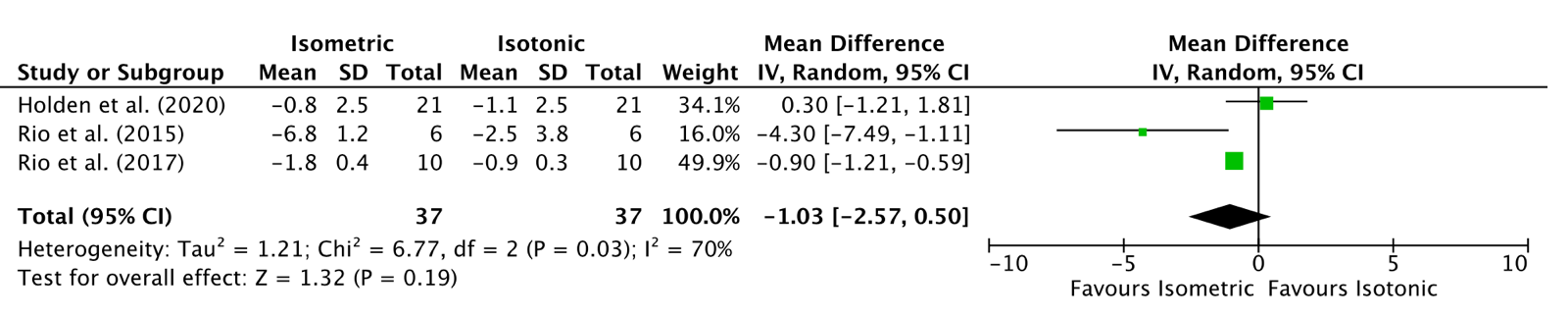
**

**Suppl. Figure S4. Meta-analysis results and forest plot of isometric exercise vs isotonic exercise for immediate post-intervention pain VAS.**

**
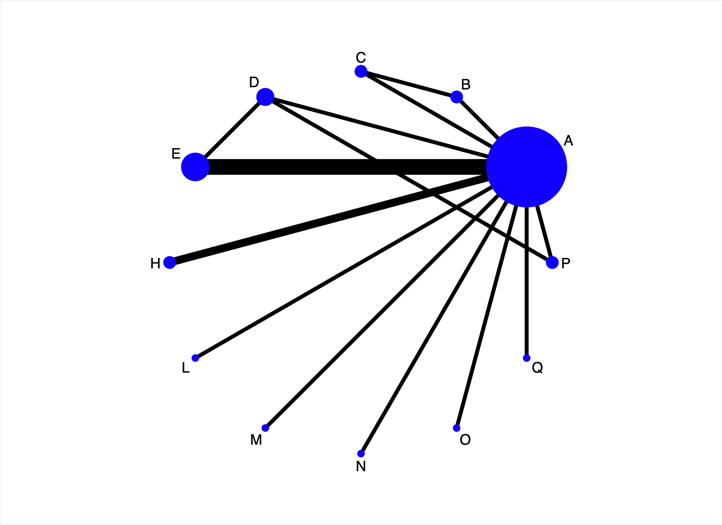
**
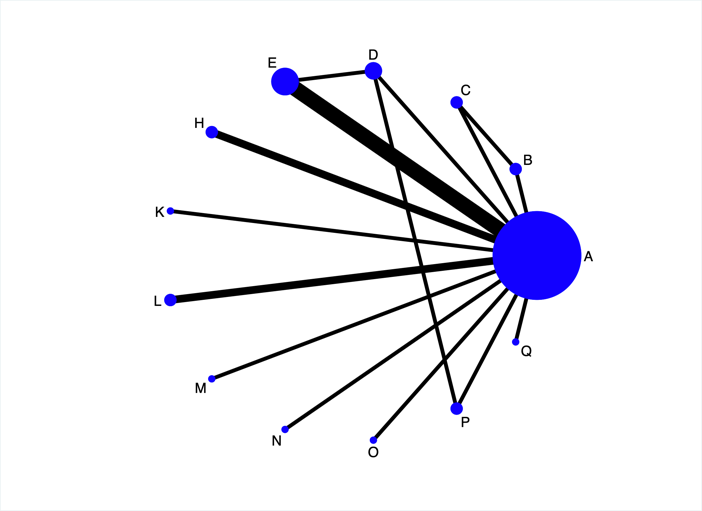


5a

5b

5c

**
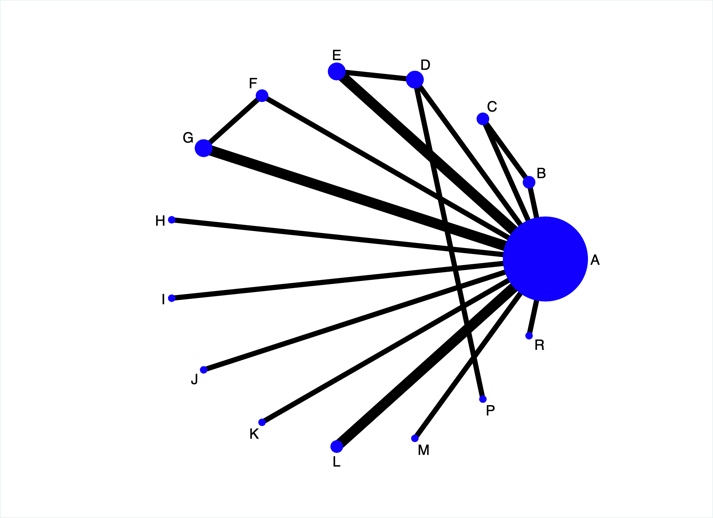

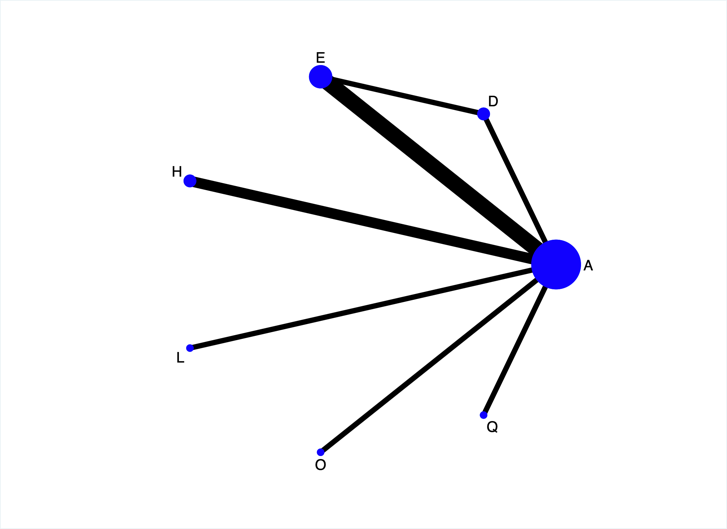
**

5d

**Suppl. Figures S5a-d. Network maps of the studies included in the network meta-analysis for short-term VISA-A (5a), mid-term VISA-A (5b), long-term VISA-A (5c) and short-term pain VAS (5d) in Achilles tendinopathy.** The size of the circle is proportional to the number of studies that represented each intervention and the thickness of the line between interventions is proportional to the number of studies assessing that comparison.

*A = Eccentric Exercise, B = MCVC + Eccentric Exercise, C = MCVC + Stretching, D = HVI (with CS) + Eccentric Exercise, E = PRP + Eccentric Exercise, F = Vibration, G = No Treatment, H = Eccentric exercise + foot orthosis, I = Stretching, J = Prolonged Tendon Loading, K = Eccentric/Concentric plus further reps, L = Low level laser therapy + Eccentric Exercise, M = Acupuncture, N = ABI + Eccentric Exercise, O = Prolotherapy + Eccentric Exercise, P = HVI (without CS) + Eccentric Exercise, Q = Heavy slow resistance training, R = heel lifts.*

**
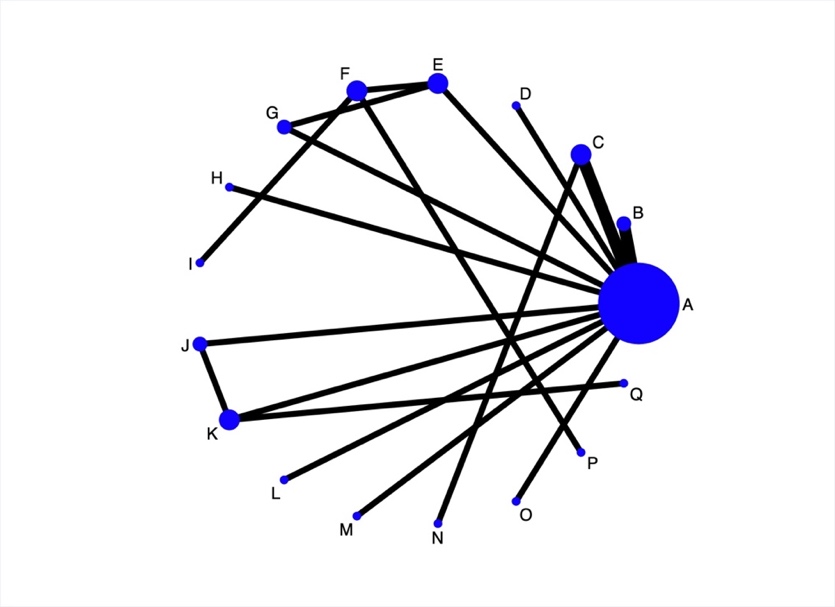
**

6b

6a

**
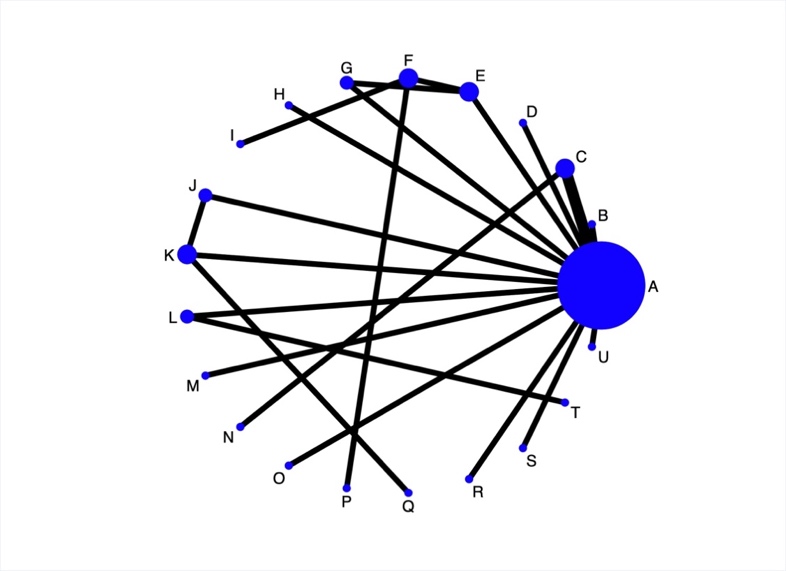
**

**Suppl. Figures S6a&b. Network maps of the studies included in the network meta-analysis for short-term pain VAS (6a) and short-term VISA-P (6b) in patellar tendinopathy.** The size of the circle is proportional to the number of studies that represented each intervention and the thickness of the line between interventions is proportional to the number of studies assessing that comparison. *A = eccentric exercise – decline squat, B = concentric exercise, C = focal extracorporeal shock-wave therapy + eccentric exercise, D = eccentric exercise - step squat, E = dry needling + eccentric exercise, F = platelet-rich plasma injection + eccentric exercise, G = percutaneous needle electrolysis + eccentric exercise, H = eccentric device, I = two platelet-rich plasma injections + eccentric exercise, J = corticosteroid injection, K = heavy slow resistance exercise, L = autologous blood injection + eccentric exercise, M = topical glyceryl trinitrate + eccentric exercise, N = radial extracorporeal shock-wave therapy + eccentric exercise, O = ultrasound therapy + eccentric exercise, P = hyaluronic acid injection + eccentric exercise, Q = moderate slow resistance exercise, R = progressive tendon loading, S = surgery, T = collagen-producing cells + eccentric exercise, U = no treatment*

**
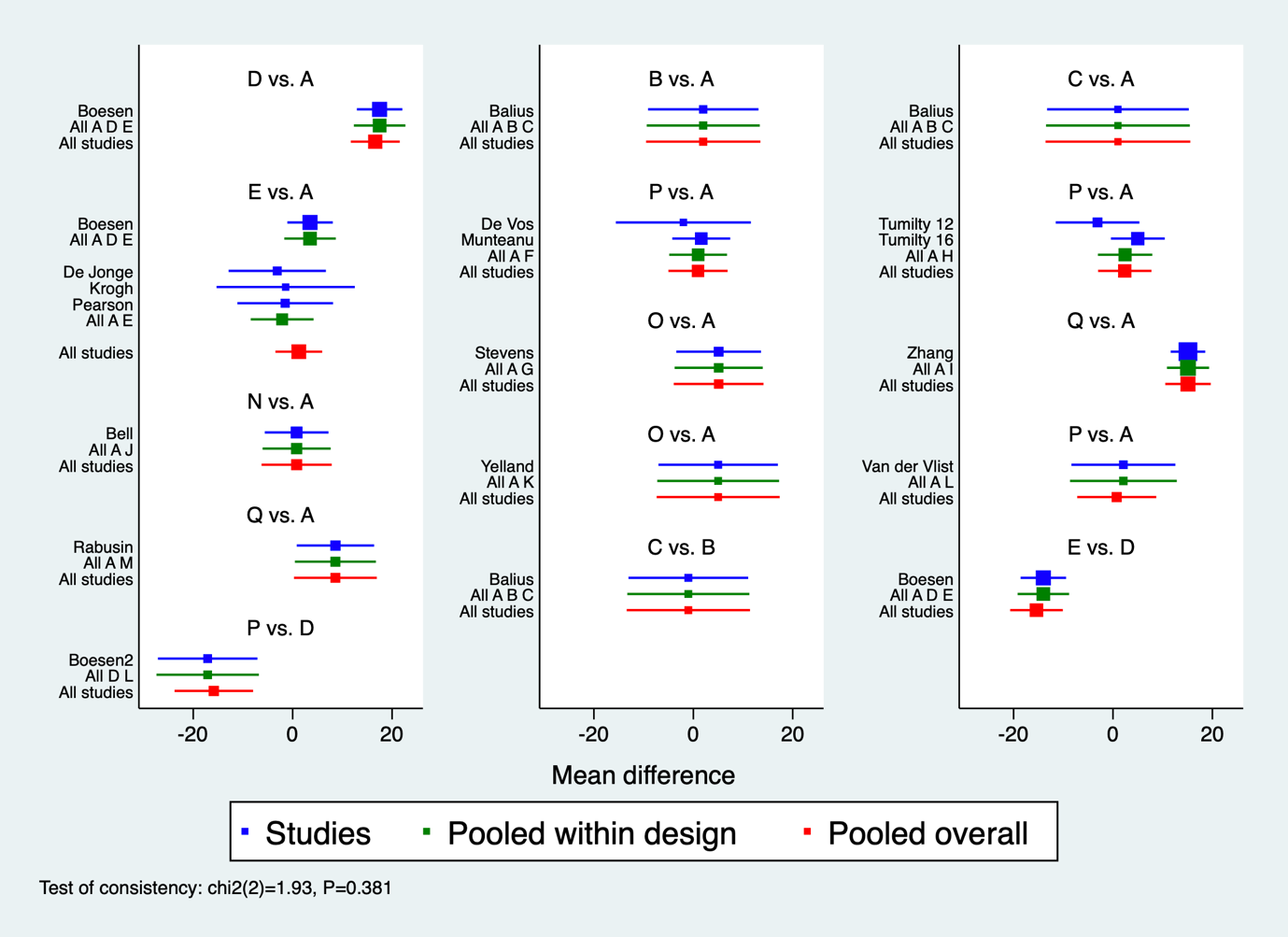
**

7a

7b


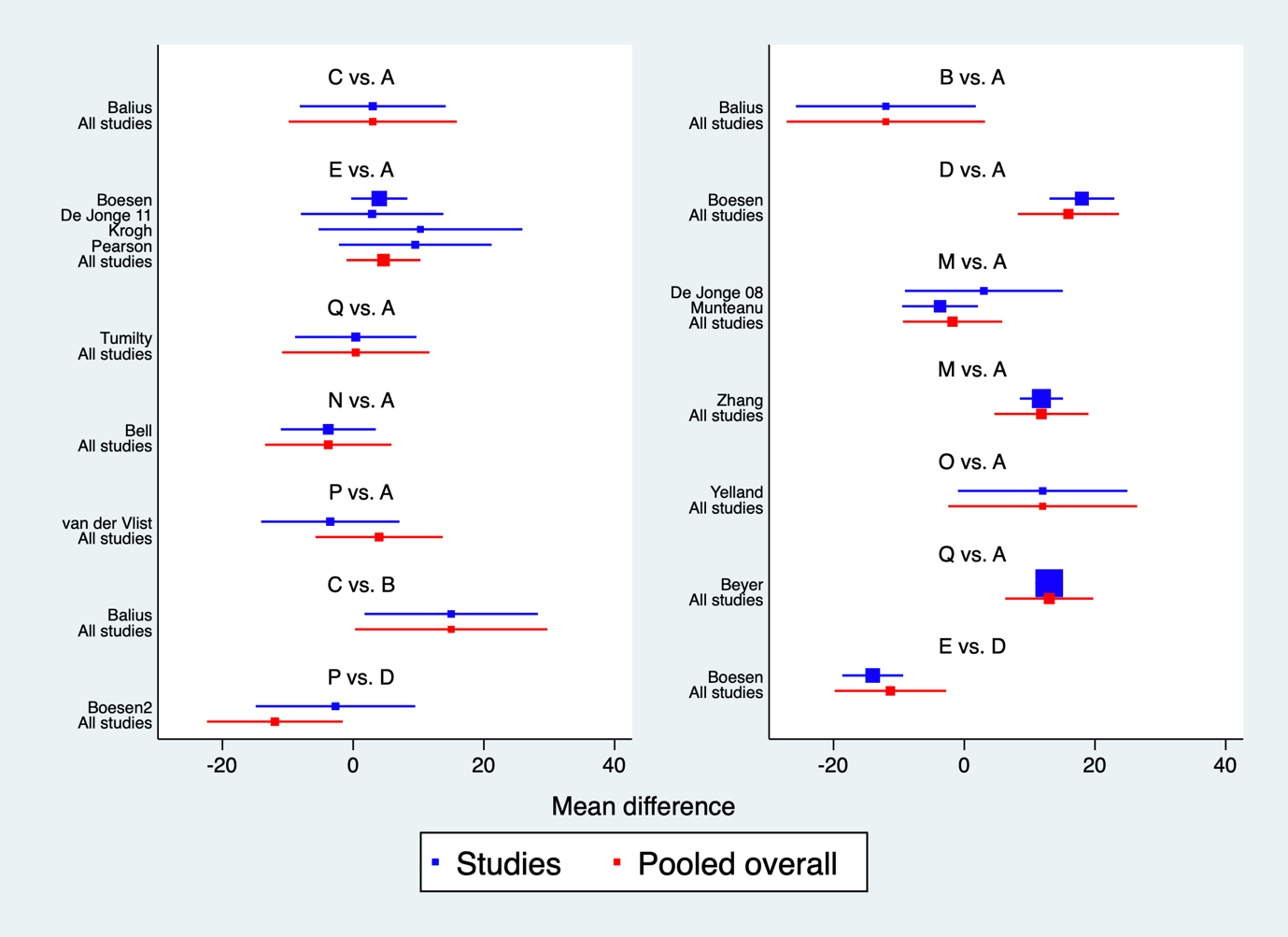


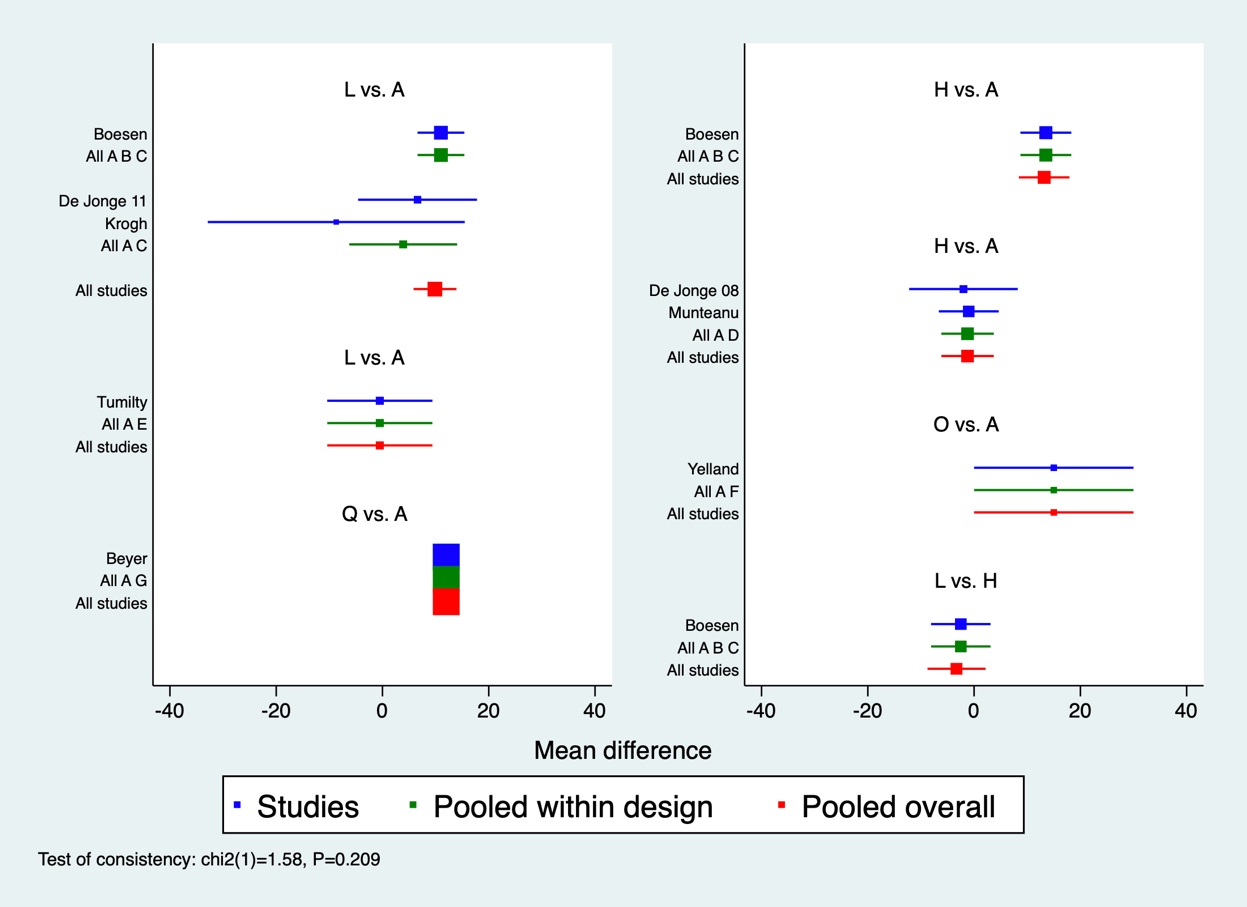


7c


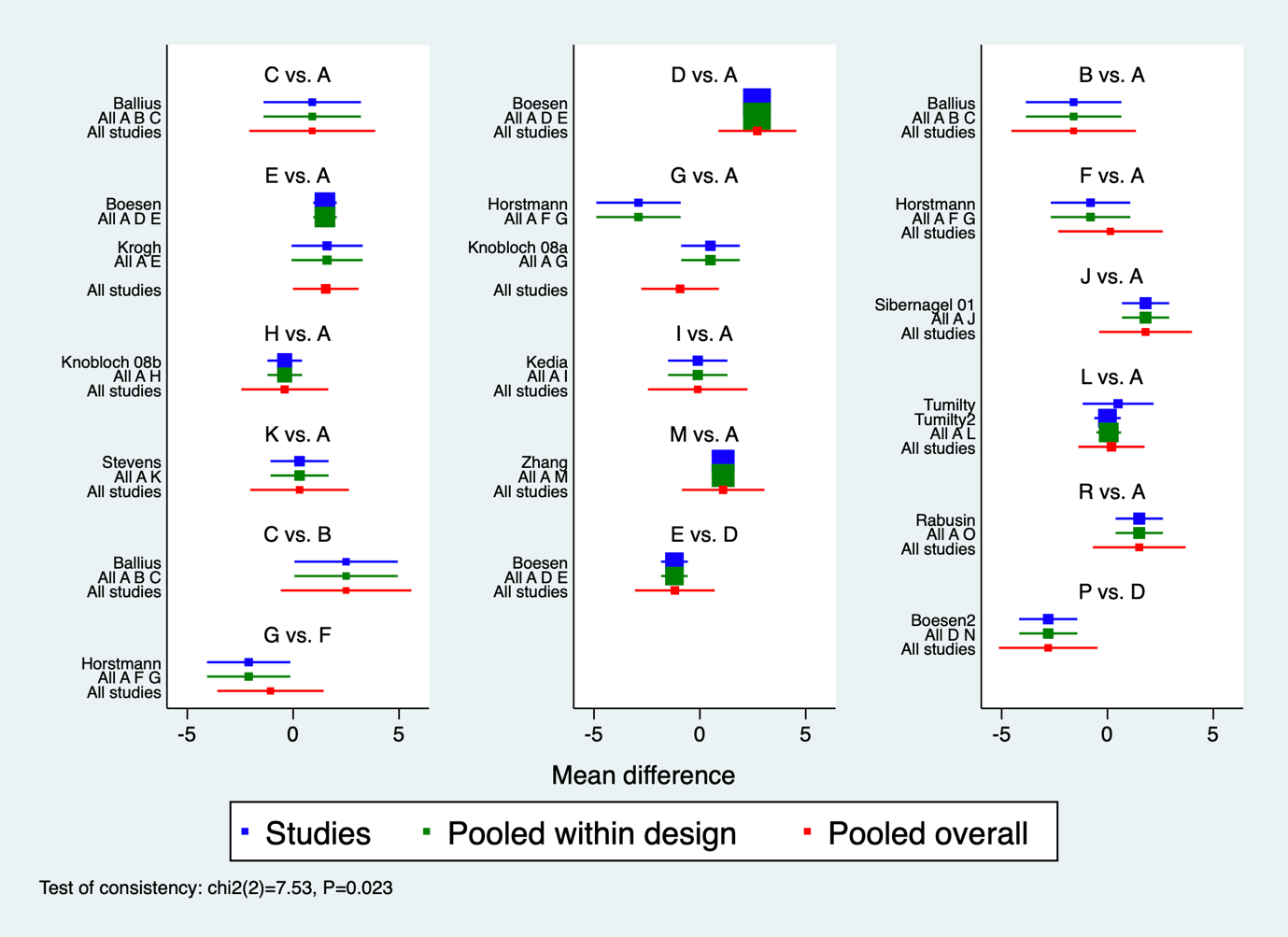


7d

**Suppl. Figures S7a-d. Network forest plots for short-term VISA-A (7a), mid-term VISA-A (7b), long-term VISA-A (7c) and short-term pain VAS (7d) in Achilles tendinopathy.** A = Eccentric Exercise, B = MCVC + Eccentric Exercise, C = MCVC + Stretching, D = HVI (with CS) + Eccentric Exercise, E = PRP + Eccentric Exercise, F = Vibration, G = No Treatment, H = Eccentric exercise + foot orthosis, I = Stretching, J = Prolonged Tendon Loading, K = Eccentric/Concentric plus further reps, L =Low level laser therapy + Eccentric Exercise, M = Acupuncture, N = ABI + Eccentric Exercise, O = Prolotherapy + Eccentric Exercise, P = HVI (without CS) + Eccentric Exercise, Q = Heavy slow resistance training, R = heel lifts.


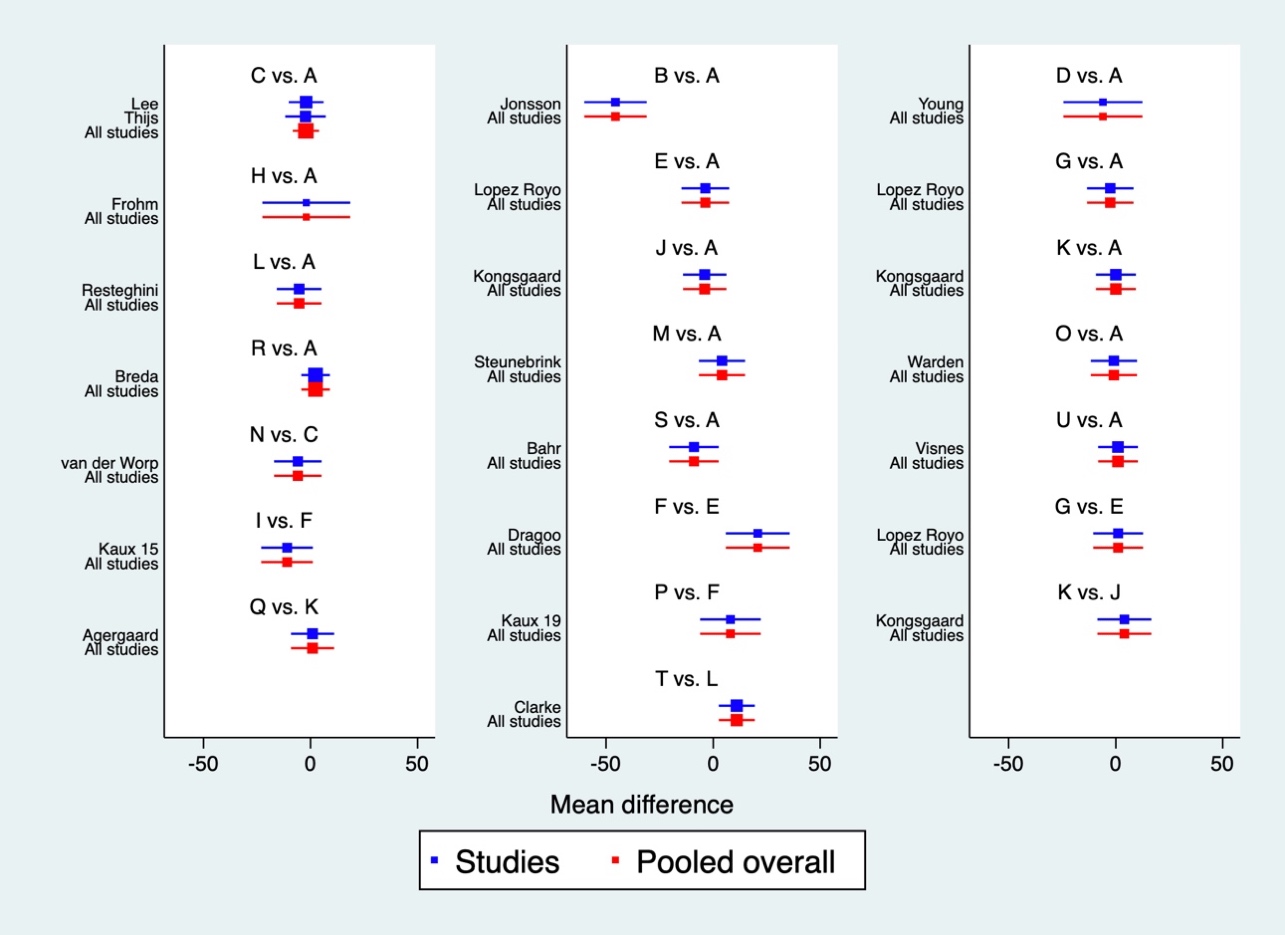


8a

8b


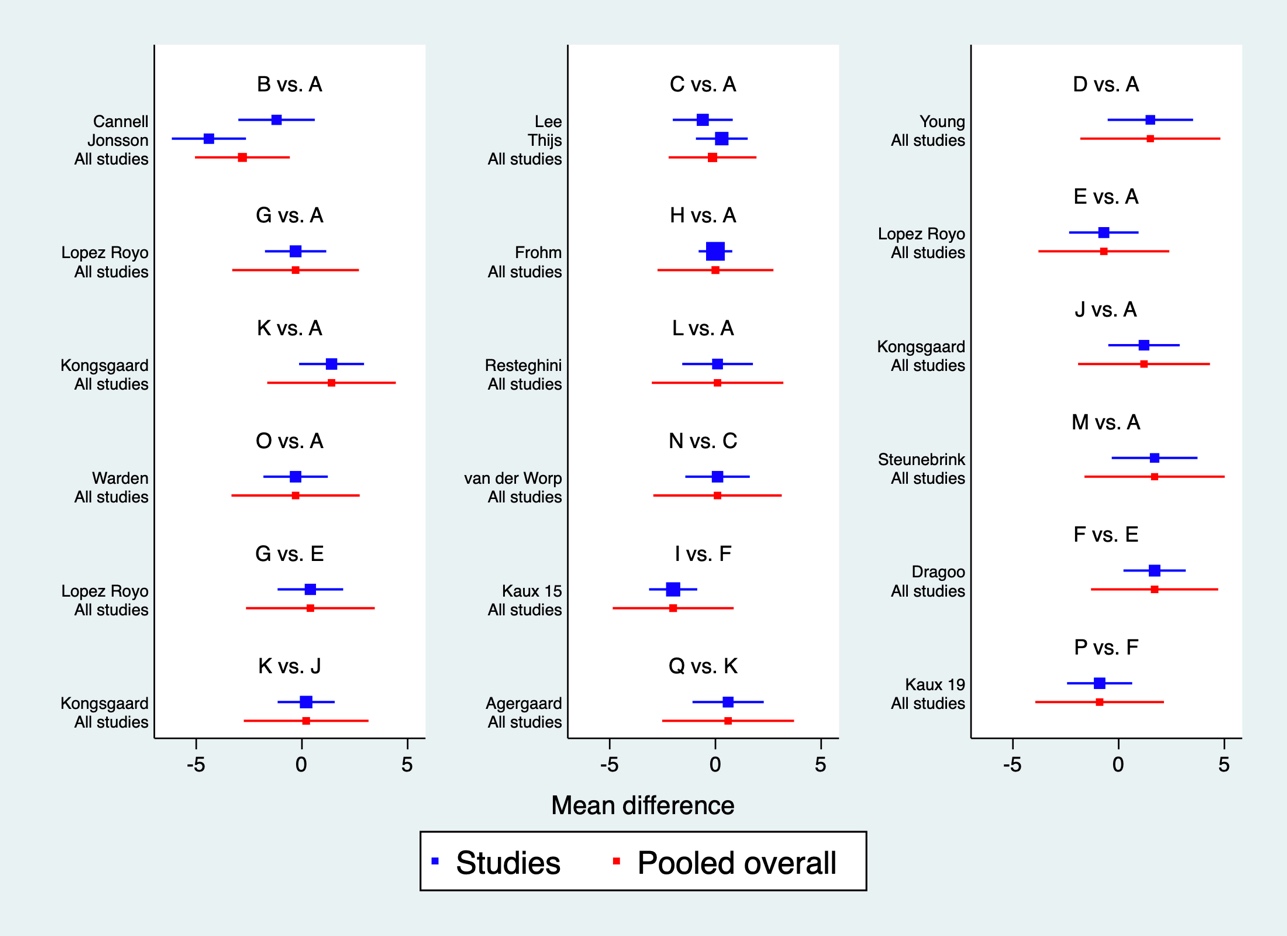


**Suppl. Figures S8a-b. Network forest plots for short-term VISA-P (8a) and short-term pain VAS (8b) in patellar tendinopathy.** *A, eccentric exercise – decline squat; B, concentric exercise; C, focal extracorporeal shock-wave therapy + eccentric exercise; D, eccentric exercise - step squat; E, dry needling + eccentric exercise; F, platelet-rich plasma injection + eccentric exercise; G, percutaneous needle electrolysis + eccentric exercise; H, eccentric device; I, two platelet-rich plasma injections + eccentric exercise; J, corticosteroid injection; K, heavy slow resistance exercise; L, autologous blood injection + eccentric exercise; M, topical glyceryl trinitrate + eccentric exercise; N, radial extracorporeal shock-wave therapy + eccentric exercise; O, ultrasound therapy + eccentric exercise; P, hyaluronic acid injection + eccentric exercise; Q, moderate slow resistance exercise; R, progressive tendon loading; S, surgery; T, collagen-producing cells + eccentric exercise; U, no treatment.*

| Eccentric Exercise +/- placebo | Eccentric Exercise + |  |  |  |  |  |  |  |  |  |  |  |
| --- | --- | --- | --- | --- | --- | --- | --- | --- | --- | --- | --- | --- |
| 12 [-2, 25] | Eccentric Exercise + MCVC |  |  |  |  |  |  |  |  |  |  |  |
| -3 [-14, 8] | **-15 [-28, -2]** | Stretching + MCVC |  |  |  |  |  |  |  |  |  |  |
| **-18 [-23, -13]** | **-30 [-45, -16]** | **-15 [-27, -3]** | Eccentric Exercise HVI (steroid) |  |  |  |  |  |  |  |  |  |
| **-5 [-8, -1]** | **-16 [-31, -3]** | -2 [-13, 9] | **14 [9, 18]** | Eccentric Exercise + PRP |  |  |  |  |  |  |  |  |
| 2 [-3, 8] | -10 [-24, 5] | 5 [-7, 18] | **21 [14, 28]** | **7 [1, 13]** | Eccentric exercise + foot orthosis |  |  |  |  |  |  |  |
| 0 [-9, 9] | -12 [-29, 4] | 3 [-11, 17] | **18 [8, 29]** | 4 [-5, 14] | -3 [-13, 7] | Eccentric Exercise + LL-LT |  |  |  |  |  |  |
| **-12 [-15, -8]** | **-24 [-37, -9]** | -9 [-20, 3] | **7 [1, 12]** | **-7 [-11, -2]** | **-14 [-20, -8]** | **-11 [-21, -2]** | Acupuncture |  |  |  |  |  |
| 4 [-3, 11] | -8 [-23, 7] | 7 [-6, 20] | **22 [13, 30]** | **9 [0.4, 17]** | 1 [-7, 10] | 4 [-8, 16] | **16 [7, 23]** | Eccentric Exercise. + AB injection |  |  |  |  |
| -12 [-24, 1] | **-24 [-43, -5]** | -9 [-26, 8] | 6 [-7, 20] | -7 [-20, 6] | **-14 [-28, -0.5]** | -12 [-27, 4] | 0 [-13, 13] | **-16 [-30, -1]** | Eccentric Exercise + Prolotherapy |  |  |  |
| 4 [-7, 14] | -8 [-25, 8] | 7 [-8, 21] | **22 [10, 33]** | 8 [-2, 19] | 1 [-10, 12] | 4 [-10, 17] | **15 [4, 26]** | 0 [-13, 13] | 15 [-1, 32] | Eccentric Exercise HVI (no steroid) |  |  |
| **-13 [-15, -10]** | **-25 [-38, -11]** | -10 [-21, 1] | **5 [0.2, 10]** | **-8 [-12, -4]** | **-15 [-21, -9]** | **-13 [-22, -3]** | -1 [-5, 2] | **-17 [-24, -9]** | -1 [-14, 12] | **-16 [-27, -5]** | Heavy slow resistance training |  |
| -9 [-17, 0] | -7 [-21, 8] | -8 [-25, 10] | 8 [-3, 18] | -8 [-18, 3] | -8 [-19, 3] | -3 [-16, 9] | -6 [-17, 4] | 7 [-4, 17] | -8 [-20, 4] | -4 [-19, 12] | -6 [-21, 8] | Heel lifts |

9a

9b

| Eccentric Exercise +/- placebo |  |  |  |  |  |  |  |  |  |  |  |
| --- | --- | --- | --- | --- | --- | --- | --- | --- | --- | --- | --- |
| 12 [-3, 27] | Eccentric Exercise + MCVC |  |  |  |  |  |  |  |  |  |  |
| -3 [-15, 9] | **-15 [-30, -1]** | Stretching +MCVC |  |  |  |  |  |  |  |  |  |
| **-16 [-22, -10]** | **-28 [-45, -11]** | **-15 [-30, -1]** | Eccentric Exercise + HVI (steroid) |  |  |  |  |  |  |  |  |
| -5 [-10, 1] | **-17 [-32, 0]** | 0 [-15, 15] | **11 [3, 19]** | Eccentric Exercise + PRP injection |  |  |  |  |  |  |  |
| 2 [-5, 9] | -11 [-27 ,7] | 0 [-16, 16] | **18 [6, 29]** | 6 [-2, 15] | Eccentric exercise + foot orthosis |  |  |  |  |  |  |
| 0 [-11, 10] | -12 [-31, 6] | -4 [-24, 16] | **16 [2, 29]** | 4 [-8, 16] | -2 [-15, 11] | Eccentric Exercise + LL-LT |  |  |  |  |  |
| **-12 [-19, -5]** | **-24 [-40, -7]** | -1 [-18, 17] | 4 [-6, 14] | -7 [-16, 2] | **-13 [-24, -3]** | -11 [-24, 2] | Acupuncture |  |  |  |  |
| 4 [-6, 13] | -8 [-26, 10] | -14 [-32, 4] | **20 [7, 32]** | 8 [-3, 19] | 2 [-10, 14] | 4 [-10, 19] | **16 [4, 28]** | Eccentric Exercise + AB injection |  |  |  |
| -12 [-26, 3] | **-24 [-44, -3]** | 0 [-19, 19] | 4 [-12, 20] | -7 [-22, 8] | -13 [-30, 2] | -12 [-30, 7] | 0 [-16, 16] | -15 [-33, 2] | Eccentric Exercise  + Prolotherapy |  |  |
| -4 [-13, 5] | -15 [-34, 2] | -4 [-25, 17] | **12 [2, 22]** | 1 [-10, 11] | -5 [-18, 6] | -4 [-18, 11] | 8 [-4, 19] | -8 [-21, 6] | 9 [-9, 25] | Eccentric Exercise HVI (no steroid) |  |
| **-13 [-19, -6]** | **-25 [-42, -8]** | -1 [-19, 17] | 3 [-7, 13] | -8 [-17, 1] | **-15 [-25, -5]** | -13 [-26, 1] | -1 [-11, 9] | **-17 [-21, -5]** | -1 [-14, 12] | -9 [-20, 2] | Heavy Slow Resistance Exercise |

9c

| Eccentric Exercise +/- placebo |  |  |  |  |  |  |
| --- | --- | --- | --- | --- | --- | --- |
| **-13 [-18, -8]** | Eccentric Exercise + HVI + steroid |  |  |  |  |  |
| **-10 [-14, -6]** | 3 [-2, 8] | Eccentric Exercise + PRP |  |  |  |  |
| 1 [-3, 6] | **14 [7, 21]** | **11 [4, 17]** | Eccentric exercise + foot orthosis |  |  |  |
| 1 [-9, 10] | **14 [3, 25]** | 10 [0, 21] | -1 [-11, 10] | Eccentric Exercise + LL-LT |  |  |
| -15 [-30, 0] | -2 [-17, 13] | -5 [-20, 10] | **-16 [-32, -0.4]** | -15 [-33, 2] | Eccentric Exercise + Prolotherapy |  |
| **-12 [-14, -10]** | 1 [-4, 6] | -2 [-6, 2] | **-13 [-18, -8]** | **-12 [-22, -2]** | 3 [-12, 18] | Heavy slow resistance training |

9d

| Eccentric Exercise +/- placebo |  |  |  |  |  |  |  |  |  |  |  |  |  |  |
| --- | --- | --- | --- | --- | --- | --- | --- | --- | --- | --- | --- | --- | --- | --- |
| -1.6 [-4.5, 1.3] | MCVC + Eccentric Exercise |  |  |  |  |  |  |  |  |  |  |  |  |  |
| 0.9 [-2.1, 3.9] | 2.5 [-0.6, 5.6] | MCVC + Stretching |  |  |  |  |  |  |  |  |  |  |  |  |
| **2.7 [0.9, 4.6]** | **4.3 [0.8, 7.8]** | 1.8 [-1.7, 5.3] | HVI (steroid) + Eccentric Exercise |  |  |  |  |  |  |  |  |  |  |  |
| 1.5 [-0.01, 3.1] | 3.1 [-0.2, 6.5] | 0.6 [-2.7, 4] | -1.2 [-3.1, 0.7] | PRP + Eccentric Exercise |  |  |  |  |  |  |  |  |  |  |
| 0.1 [-2.3, 2.6] | 1.7 [-2.1, 5.6] | -0.8 [-4.6, 3.1] | -2.6 [-5.7, 0.5] | -1.4 [-4.3, 1.5] | Vibration |  |  |  |  |  |  |  |  |  |
| -0.9 [-2.8, 0.9] | 0.7[-2.8, 4.1] | -1.8 [-5.3, 1.7] | **-3.6 [-6.2, -1]** | **-2.5 [-4.9, -0.6]** | -1.1 [-3.6, 1.4] | No Treatment |  |  |  |  |  |  |  |  |
| -0.4 [-2.4, 1.6] | 1.2 [-2.4, 4.8] | -1.3 [-4.9, 2.3] | **-3.1 [-5.9, -0.4]** | -1.9 [-4.5, 0.6] | -0.5 [-3.8, 2.7] | 0.5 [-2.2, 3.3] | Eccentric exercise + foot orthosis |  |  |  |  |  |  |  |
| -0.1 [-2.4, 2.3] | 1.5 [-2.3, 5.3] | -1 [-4.5 ,2.8] | -2.8 [-5.8, 0.2] | -1.6 [-4.4, 1.2] | -0.2 [-3.7, 3.2] | 0.8 [-2.2, 3.8] | 0.3 [-2.8, 3.4] | Stretching |  |  |  |  |  |  |
| 1.8 [-0.4, 4] | 3.4 [-0.3, 7.1] | 0.9 [-2.8, 4.6] | -0.9 [-3.8, 1.9] | 0.3 [-2.4, 2.9] | 1.7 [-1.6, 5] | 2.7 [-0.1, 5.6] | 2.2 [-0.8, 5.2] | 1.9 [-1.3, 5.1] | Prolonged Tendon Loading |  |  |  |  |  |
| 0.3 [-2, 2.6] | 1.9 [-1.9, 5.7] | -0.6 [-4.4 ,3.2] | -2.4 [-5.4, 0.6] | -1.2 [-4, 1.6] | 0.2 [-3.2, 3.6] | 1.2 [-1.7, 4.2] | 0.7 [-2.4, 3.8] | 0.4 [-2.9, 3.7] | -1.5 [-4.7, 1.7] | Eccentric/Concentric plus further reps |  |  |  |  |
| 0.2 [-1.4, 1.8] | 1.8 [-1.5, 5.1] | -0.7 [-4.1, 2.7] | **-2.5 [-4.9, -0.1]** | -1.3 [-3.6, 0.8] | 0.1 [-2.9, 3] | 1.1 [-1.3, 3.6] | 0.6 [-2, 3.2] | 0.3 [-2.5, 3.1] | -1.6 [-4.3, 1.1] | -0.1 [-2.9, 2.7] | LL-LT + Eccentric Exercise |  |  |  |
| 1.1 [-0.8, 3] | 2.7 [-0.8, 6.2] | 0.2 [-3.4, 3.8] | -1.6 [-4.3, 1.1] | -0.4 [-2.9, 2.1] | 1 [2.2, 4.1] | 2 [-0.6, 4.7] | 1.5 [-1.3, 4.3] | 1.2 [-1.9, 4.3] | -0.7 [-3.6, 2.2] | 0.8 [-2.2, 3.8] | 0.9 [-1.6, 3.4] | Acupuncture |  |  |
| 1.5 [-0.7, 3.7] | 3.1 [-0.6, 3.8] | 0.6 [-3.1, 4.3] | -1.2 [-4.1, 1.6] | 0 [-2.7, 2.7] | 1.4 [-1.9, 4.7] | 2.4 [-0.4, 5.3] | 1.9 [-1.1, 4.9] | 1.6 [-1.6, 4.8] | -0.3 [-3.4, 2.8] | 1.2 [-2, 4.4] | 1.3 [-1.4, 4] | 0.4 [-2.5, 3.3] | Heel lifts |  |
| -0.1 [-3.1, 2.9] | 1.5 [-2.7, 5.7] | -1 [-5, 3.2] | **-2.8 [-5.1, -0.5]** | -1.6 [-4.6, 1.4] | -0.2 [-4.1, 3.6] | 0.8 [-2.6, 4.3] | 0.3 [-3.3, 3.9] | 0 [-3.7, 3.8] | -1.9 [-5.6, 1.8] | -0.4 [-4.2, 3.4] | -0.3 [-3.6, 3.1] | -1.2 [-4.8, 2.4] | -1.6 [-5.3, 2.1] | HVI (no steroid) + Ecc Ex |

**Suppl. Figures S9a-d. Comparative treatment class effects expressed as mean difference with 95% confidence interval for VISA-A at short-term (9a), mid-term (9b) and long-term (9c) follow-up and for pain VAS at short-term follow up (9d) in Achilles tendinopathy.** Each cell represents the result of the comparison of the intervention of that column vs the intervention of that row. A negative value in a cell favours the row intervention in VISA-A treatment class effects and the column intervention in pain VAS treatment class effects. Bold indicates statistical significance. *AB; Autologous blood injection; HVI, High volume injection; LL-LT, Low-level laser therapy; MVCS, mucopolysaccharides, type I collagen injection plus vitamin supplementation.*

*
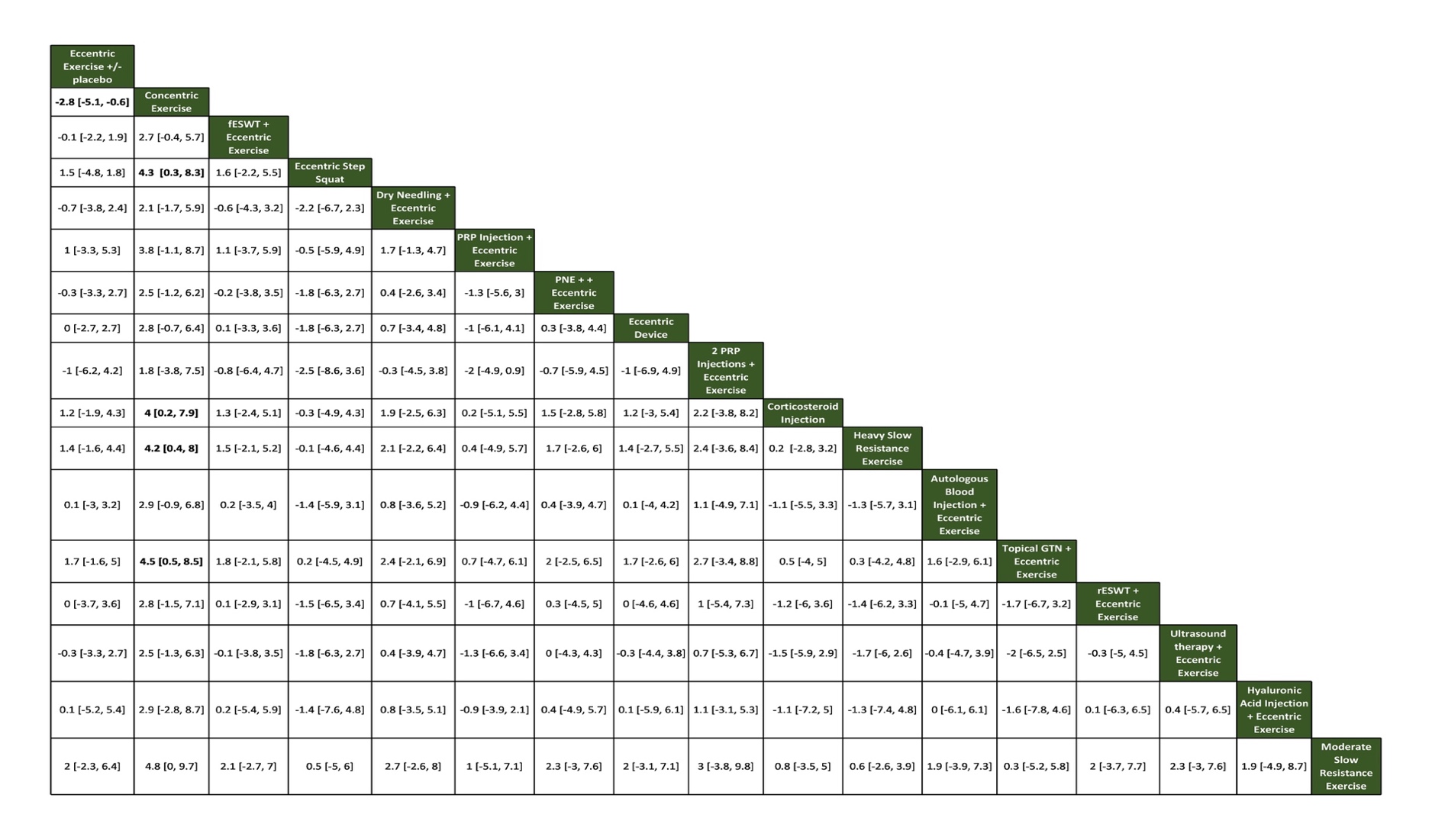
*

10a

*
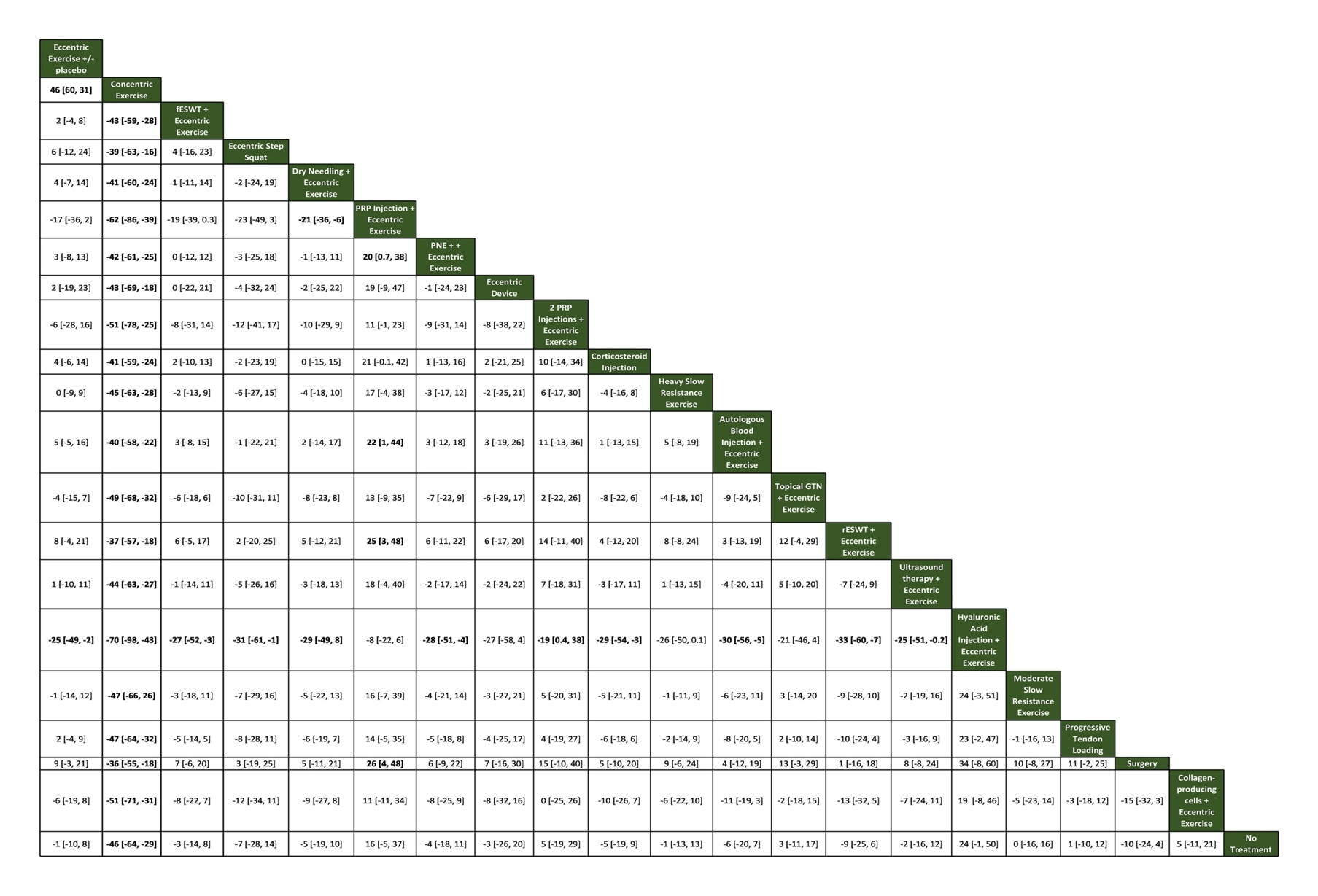
*

10b

**Suppl.** **Figures S10a-b. Comparative treatment class effects expressed as mean difference with 95% confidence interval for short-term pain VAS (10a) and VISA-A (10b).** Each cell represents the result of the comparison of the intervention of that column vs the intervention of that row. A negative value in a cell favours the column intervention in VISA-A treatment class effects and the column intervention in pain VAS treatment class effects. Bold indicates statistical significance. *GTN, glyceryl trinitrate; ESWT, extracorporeal shock wave therapy; PNE, percutaneous needle electrolysis; PRP, platelet-rich plasma.*

**Supplementary Tables**

| Supplementary Table S1. Achilles Tendinopathy | | | | |  |  |  |
| --- | --- | --- | --- | --- | --- | --- | --- |
| Study | Population (mean age) | Mean DOS (range) | Interventions | Duration of treatment | Follow up (weeks) | Pain score | Function score |
| Balius et al  (2016) | N=59 (40.8yr) | Not recorded | 1)Eccentric exercise (N=19).  2)Eccentric exercise + MCVC (N=19).  3)Passive stretching + MCVC (N=20)  *(MCVC) mucopolysaccharides, type I collagen, and vitamin C supplementation.  **Alfredson et al – eccentric exercise programme. | 12 weeks | 0, 5, 12w | VAS | VISA-A |
| Bell et al (2013) | N=53 (50yr) | 30.98m | 1)AB injection + Eccentric Calf training programme – 12 weeks (N=26)  2)Needling + Eccentric Calf training programme – 12 weeks (N=27)  *. 12-week exercise programme, 180 eccentric heel drops per day, pain limited to >4/10  **Two unguided peritendinous injections at site of maximal tenderness. | 12 weeks | 0, 4, 8, 12, 24w | - | VISA-A |
| Beyer et al (2015) | N=58 (48yr) | 28.2m | 1)Eccentric training programme (N=25).  2)Heavy slow resistance training(N=23).  *Alfredson et al eccentric exercise programme 7 days a week, 12 weeks.  ** Kongsgaard et al, heavy slow resistance training, progressive load with reducing frequency – 12 weeks. | 12 weeks | 0, 12, 52w | VAS | VISA-A |
| Boesen et al (2017) | N=60 (40.4yr) | 27.5m | 1) HVI (steroid, saline, and local anaesthetic) + eccentric exercise (N=20).  2) 4 x PRP injections each 2 weeks apart + eccentric exercise (N=20).  3) placebo (a few drops of saline under the skin) + eccentric exercise (N=20). | 12 weeks | 0, 6, 12, 24w | VAS | VISA-A |
| Boesen et al (2019) | N=28 (43y) | 44w | 1)HVI (steroid, saline and local anaesthetic) + eccentric exercise (N=14).  2)HVI (saline and local anaesthetic) + eccentric exercise (N=14). | 12 weeks | 0, 6, 12, 24w | VAS | VISA-A |
| Bradford et al. (2021) | N=11 (?y) | Not identified | 1)Isometric plantarflexion exercises with knee extended (N=11)  2) Isometric plantarflexion exercises with knee flexed (N=11)  (Cross-over study) | Single bout | 0, post-intervention | VAS | - |
| De Jonge et al (2010) | N=54 (49.7yr) | 5.2 m | 1. PRP injection + eccentric exercise(N=27).  2. injection saline + eccentric exercise(N=27). | 12 weeks | 0, 6, 12, 52w | - | VISA-A |
| De Jonge et al. (2008) | N=70 (44.6yr) | 30.7m | 1)Eccentric exercise (N=34).  2) Eccentric exercise + splint (N=36) De Vos (2007) eccentric exercise protocol. | 12 weeks- | 0, 8, 52w | - | VISA-A |
| De Vos et al.  (2007) | N=63 (45.3yr) | 30.7m | 1) Eccentric exercise group.  2) Night splint group. + eccentric exercise group. | 12 weeks | 0, 12w | - | VISA-A |
| Horstmann et al.  (2013) | N=58 (45.4yr) | 31.6m | 1) Vibration training (23).  2) Eccentric training (19).  3) Wait and see (16).  *Vibration training - *Galileo Fit, 13-18 Hz.*  **12-week programme, 36 sessions, 3 x15 + additional set if symptoms allowed. | 12 weeks | 0, 12w | VAS | - |
| Herrington et al  (2007) | N=25 (36.8yr) | 24.32m | 1) Deep frictional massage, Ultrasound frequency of 1 MHz. (6 sessions) (N=13).  2) Deep frictional massage, Ultrasound frequency of 1 MHz. (6 sessions) + eccentric loading programme (N=12).  *DFM + US – 6 sessions in total (6 weeks) | 12 weeks | 0, 4, 10w | - | VISA-A |
| Knobloch, et al (2007) | N=20 (32.8yr) | Not recorded | 1)Eccentric exercise programme (N=15).  2)Control group (N=5)  *Eccentric exercise 3 x 15 – 12 weeks.  **Control group – ice symptom relief/ rest. | 12 weeks | 0, 52w | VAS | - |
| Knobloch et al (2008) | N=116 (47.5yr) | 3m | 1) Eccentric exercise (3 x 15 sets) + Air heel brace (N=57)  2) Eccentric exercise (3 x 15 sets)  (N=59)  (*Eccentric training 3x15 sets, twice daily for 12 weeks).  ** Air Heel™ Brace Vista, CA, USA). | 12 weeks | 0, 52w | VAS | - |
| Krogh et al (2016) | N=24 (49.2yr) | 45m | 1)Platelet Rich Plasma + eccentric training (N=12).  2)Saline (placebo) + eccentric training (N=12)  *Ultrasound guided injection, 6ml PRP.  **Eccentric exercise programme Fredberg et al. | 12 weeks | 0, 12, 26w | VAS | VISA-A |
| Mafi et al (2001) | N=44 (48.2yr) | 20.5m | 1)Eccentric training(N=22).  2)Concentric training(N=22).  Eccentric exercise + concentric, twice daily 7 days a week for 12 weeks, 3x15.  (As described) | 12 weeks | 0, 12w | VAS | VISA-A |
| Munteanu et al (2015) | N=140, (43.5yr) | 29.3m | 1)Customised foot orthoses N=73.  2)Sham foot orthoses + Eccentric training N=67.  * Alfredson et al eccentric exercise programme. | 12 weeks | 0, 4, 12, 26, 52w | VAS | VISA-A |
| Petersen et al (2007) | N=100 (42.5yr) | 7.4m | 1)Eccentric training (N=37).  2)Airheel brace (N=35).  3)Eccentric training + Airheel brace(N=28).  * Alfredson et al eccentric exercise programme. | 12 weeks | 0, 6, 12, 56w | VAS | - |
| Pearson et al  (2012) | N=40 (50yr) | 11m | 1)PRP injection + Eccentric (N=20)  2) Eccentric programme (N=20)  *3ml venous blood, immediately paratendon injection post venepuncture.  **Alfredson et al eccentric exercise programme. | 12 weeks | 0, 6, 12w | - | VISA-A |
| Rabusin et al. (2021) | N=100 (45.9y) | 34.4m | 1)Eccentric exercise (N=40)  2)Heel lifts (N=40) | 12 weeks | 0, 2, 6, 12w | VAS | VISA-A |
| Rompe et al (2007) | N=75 (48.6yr) | 10.8m | 1)Eccentric loading(N=25).  2)Repetitive low energy ESWT(N=25).  3)Wait and see (N=25).  *Alfredson eccentric exercise programme, 3 x15, twice daily, 12 weeks.  ** ESWT, three sessions, weekly interval. 2000 pulses with a pressure of 3 bars.  ***W&S, Stretching, simple analgesia. | 12 weeks | 0, 6, 16w | - | VISA-A |
| Rompe et al  (2009) | N=68 | 14.5m | 1)Eccentric exercise(N=34).  2)Eccentric exercise + low energy shock wave therapy (N=34).  *Alfredson et al, eccentric programme.  **SWT – 3 sessions, completed after week 4. | 12 weeks | 0, 6, 16w, | VAS | VISA-A |
| Roos et al (2003) | N=44 (45yr) | 5.5m | 1)Eccentric group (N=16).  2)Eccentric + splint group (N=15).  3)Splint group (N=13).  *Alfredson et al – eccentric exercise programme.  **Anterior splint holding foot at 90degrees. | 12 weeks | 0, 6, 12, 26, 52w | - | - |
| Silbernagel et al (2007) | N=38 (46yr) | 36.2m | 1)Exercise training group (N=19)  (eccentric to concentric progression)  2)Active rest group – 6 weeks + engage in exercise (N=19)  *Achilles tendon loading for the first 6 weeks of rehabilitation.  **Not allowed to perform the physical activity for 6 weeks.  *Exercise protocol: 2 legged, 1 legged, eccentric and toe raises. Progressive exercise over 12 weeks based on patient status. | 6 weeks rest control group.  Exercise 36 weeks | 0, 6, 12, 24, 52w | VAS | VISA-A |
| Silbernagel et al (2001) | N=32  (40 tendons) (45yr) | 36m | 1)Exercise group – progressive loading (N=22).  2)Control group (N=18).  * Concentric/eccentric exercise + unilateral eccentric exercise– progressive overload training regime (12 weeks)  **Concentric/eccentric exercise – standard – up to 3 sets 5 repetitions not allowed to progress to pain. | 12 weeks | 0, 6, 12, 52w | VAS | - |
| Stevens et al  (2013) | N=28 (48.6yr) | 7.4m | 1)Eccentric exercise Alfredson protocol (N=15)  2)Eccentric exercise Alfredson protocol + further repetition that was tolerable (prescription not defined) (N=14)  Alfredson 180 reps per day.  3x15 rep, two training positions. | 6 weeks | 0, 3, 6w | VAS | VISA-A |
| Tumilty et al  (2016) | N=80 (47yr) | 3m | 1)Laser-induced photobiomodulation + exercise regime 1. (N=20)  2)Laser-induced photobiomodulation + exercise regime 2. (N=20)  3)Placebo + exercise regime 1 (N=20)  **4)Placebo + exercise regime 2. (N=20)**  ER 1. Eccentric heavy load training, BD – 7 days.  ER 2 Eccentric heavy load training Twice a week. | Exercise protocol – 12 weeks.  Laser protocol two times per week with 48 hours between applications. | 0, 4, 12w | VAS | VISA-A |
| Tumilty et al (2012) | N=40 (46.1yr) | Not recorded | 1)Low level laser therapy + eccentric exercise (N=20).  2) Low level laser therapy + placebo (N=20).  *810nm, 18J per session. 3 times per week for 4 weeks.  **Alfredson protocol. | 12 weeks | 0, 4, 12, 52w | VAS | VISA-A |
| Van der Vlist et al (2020a) | N=80  (47.9yr) | 46m | 1)High volume injection 5x 10ml syringe (8ml 0.9 NaCl + 2ml 2% lidocaine33 ) + eccentric exercise  2)Needling, injection 2ml saline + lidocaine mixture + eccentric exercise . | 12 weeks | 0, 2, 6, 12, 24w | VAS | VISA-A |
| Van der Vlist et al (2020b) | N=91 (48y) | 59-104w median | 1)Isometric exercise (tiptoes) (N=24)  2)Isometric exercise (dorsiflexed ankle) (N=18)  3)Isotonic exercise (N=24)  4)Rest (N=25) | Single bout | 0, post-intervention | VAS | - |
| Yelland et al  (2009) | N=43 (46yr) | 17.4m | 1)Eccentric exercise(N=15).  2) Prolotherapy injections (N=14)  3)Eccentric exercise + prolotherapy (N=14)  *Alfredson et al – exercise programme. 12 weeks limited by 4/10 pain tolerance.  **Sub cut injection of glucose 0.1%, lignocaine 0.1%, weekly from week 4-12. | 12 weeks | 0, 6, 12, 26, 52w | - | VISA-A |
| Zhang et al (2013) | N=64 (51yr) | 17m | 1)Eccentric exercise (N=32)  2)Acupuncture treatments (N=32)  *Eccentric training 2(+1 if tolerated) x 15.  **Acupuncture, three times per week, 30 minutes duration for 8 weeks. | 8 weeks | 0, 8, 15,24w | VAS | VISA-A |

**Suppl. Table S1. Characteristics of the included randomised controlled trials for Achilles Tendinopathy (participants, interventions, comparators and outcome measures).** *ESWT, extracorporeal shock-wave therapy; months; HA, hyaluronic acid; LP-PRP; LR-PRP, leucocyte-rich PRP; NRS, numerical rating scale; PRP, platelet-rich plasma; US, ultrasound; VAS, visual analogue scale; VISA-P, Victorian Institute of Sports Assessment – Patellar; w, weeks; y, years.*

| Supplementary Table S2. GTPS | | | |  |  |  |  |
| --- | --- | --- | --- | --- | --- | --- | --- |
| Study | Population (mean age) | Mean DOS (range) | Interventions | Duration of treatment | Follow up Months | Pain score | Function score |
| Mellor et al (2018) | N=204 (55yr) | Not recorded | 1)Education + exercise(N=69).  2)US-guided CSI(N=66).  3)Wait + see(N=69).  *Exercise: 14 session education over 8 weeks, focused on strengthening hip abductors. 4-6 exercises per day. | 8 weeks exercise  Single CSI | 0, 8, 12, 26, 52w | VAS | VISA-G |
| Rompe et al (2009) | N=213 (48yr) | 13.1 | 1)Home training (N=76)  2)CSI (N=75).  3)r-ESWT (N=78)  *progressive slow repetitive exercises.  Iliotibial band stretch standing  Piriformis stretching.  Straight leg raises.  Wall squat with ball  Gluteal strengthening  **3 sessions of ESWT, return to normal activity levels after 6 weeks. | 12 weeks exercise  Single CSI  3 weekly ESWT sessions | 0, 6, 52w | VAS | - |
| Ganderton et al (2018) | N=94 (62yr) | Not recorded | 1)Exercise Group (GLOBE) (N=46)  2)Sham Group (N=48)  *Exercise group - isometric loading of the gluteus Medius and minimums, and kinetic chain  **Sham seated exercises not aimed at therapeutic loading of the gluteal tendons or strengthening of the kinetic chain. | 12 weeks | 0, 12, 52w | - | VISA-G |
| Clifford et al (2019) | N=30 (59yr) | 22.95m | 1)Isometric exercise programme (N=15)  2)Isotonic exercise programme (N=15)  *Hip abduction hold – 30 seconds. Standing gluteal contraction. 3 x10, daily.  **Controlled hip abduction, standing hip abduction slide. | 12 weeks | 0, 4, 12w | VAS | VISA-G |
| Heaver et al. (2021) | N=104 (62yr) | Not recorded | 1) f-ESWT + exercise (N=53)  2) US-guided CSI + exercise (N=51)  *Home exercise programme consisting of progressive slow repetitive exercises. | Single CSI  3 ESWT treatments over 2w  Exercise duration not specified | 0, 3, 12m | VAS | - |
| Ramon et al. (2020) | N=103 (56yr) | Not recorded | 1) f-ESWT + exercise (N=53)  2) Sham f-ESWT + exercise (N=50)  *Home exercise programme consisting of progressive slow repetitive exercises. | 3 weekly ESWT sessions  24 weeks of exercise | 0, 2m | VAS | - |

**Suppl. Table S2. Characteristics of the included randomised controlled trials for GTPS (participants, interventions, comparators and outcome measures).** *CSI, corticosteroid injection;* *f-ESWT, focal extracorporeal shock-wave therapy; months; r-ESWT, radial ESWT; US, ultrasound; VAS, visual analogue scale; VISA-G, Victorian Institute of Sports Assessment – Gluteal; w, weeks; y, years.*

| Supplementary Table S3. Patellar Tendinopathy | | | |  |  |  |  |
| --- | --- | --- | --- | --- | --- | --- | --- |
| Study | Population (mean age) | Mean DOS (range) | Interventions | Duration of treatment | Follow up | Pain score | Function score |
| Abat et al. (2016) | N=64 (31yr) | 29.2m | 1)Electro-physiotherapy + eccentric exercise n=32  2)US-guided galvanic electrolysis + eccentric exercise n=32 | 8 weeks | 0, 8w | - | VISA-P |
| Agergaard et al. (2021) | N=44 (31yr) | 7.1m (3-12m) | 1)Moderate slow resistance exercise  2)Heavy slow resistance exercise | 12 weeks | 0, 6w, 12w, 52w | VAS | VISA-P |
| Bahr et al (2006) | N=35 (40 tendons) (31yr) | 33m (6-100m) | 1)Eccentric exercise (decline squat) n=20 tendons  2)Surgery n=20 tendons | Exercise for 12 weeks min | 0, 12w, 26w, 52w | VAS | VISA-P |
| Breda et al (2020) | N=76 (24yr) | 98.5m | 1) PTLE Progressive tendon loading exercises.  2)Eccentric exercise therapy | 12 weeks | 0, 12w, 24w. | - | VISA-P |
| Cannell et al (2001) | N=19 (26yr) | 3.6m (1.6-6.1m) | 1)Eccentric exercise (drop squat) n=10  2)Concentric exercise n=9 | 12 weeks | 0, 6w, 12w | VAS | - |
| De Vries et al. (2016) | N=97 (27yr) | 18m (?m-?m) | 1)Patellar strap n=21  2)Sports taping n=18  3)Placebo taping n=16  4)No treatment n=14 | 1 week | 0, 1w | VAS | - |
| Dragoo et al. (2014) | N=23 (35yr) | Not stated | 1)Dry needling + eccentric exercise n=13  2)PRP + eccentric exercise n=10 | Single treatment with dry needling and PRP  Eccentric programme for duration of study | 0, 3w, 6w, 9w, 12w, ≥26w | VAS | VISA-P |
| Frohm et al (2007) | N=20 athletes (27yr) | Not stated (>3m) | 1)Eccentric device (Bromsman) n=11  2)Eccentric exercise (decline squat) n=9 | Supervised eccentric exercise twice weekly for 12 weeks | Weekly. Isokinetic tests 0, 12w | VAS | VISA-P |
| Holden et al. (2020) | N=21 (26.5yr) | 24m (10-84m) | 1) Isometric exercise n=21  2) Dynamic (isotonic) exercise n=21  Same group performed both interventions (cross-over) | Single session of each intervention | 0, post-intervention | VAS | - |
| Jonsson et al (2005) | N=19 tendons (15 patients) (24.9yr) | 17.4m (8-72m) | 1)Eccentric exercise (decline squat) n=10 tendons  2)Concentric exercise n=9 tendons | Exercise daily for 12 weeks | 0, 12w | VAS | VISA-P |
| Kaux et al (2015) | N=20 (30.3yr) | 17m | 1)Single PRP injection + eccentric exercise n=10  2) Two PRP injections + eccentric exercise n=10 | Single injection or two injections within a week | 0, 6w, 3m, 12m | VAS | VISA-P |
| Kongsgaard et al. (2009) | N=37 (32.4yr) | 18.3m  (>3-36m) | 1)Corticosteroid injection n=12  2)Eccentric exercise n=12  3)Heavy slow resistance exercise n=13 | 12 weeks | 0, 12w, 24w | VAS | VISA-P |
| Lee et al (2017) | N=34 (22.6yr) | 33.6m | 1)f-ESWT + eccentric exercise n=17  2)Sham ESWT + eccentric exercise n=17 | 6 sessions of ESWT over 6 weeks  Eccentric exercise for 12 weeks | 0, 12w | VAS | VISA-P |
| Lopez-Royo et al. (2021) | N=50 athletes (32.5y) | >3m | 1) Dry needling + eccentric exercise n=16  2) Percutaneous needle electrolysis + eccentric exercise n=17  3) Eccentric exercise n=17 | 4 needling sessions over 8 weeks | 0, 10w, 22w | VAS | VISA-P |
| Resteghini et al. (2016) | N=22 (38.9yr) | 18m (5-36m) | 1)Autologous Blood Injection + eccentric exercise n=11  2)Placebo injection + eccentric exercise n=11 | Single injection | 0, 1m, 3m, 12m | VAS | VISA-P |
| Rigby et al. (2015) | N=31 (24.6yr) | >1m  <2y | 1)Wired iontophoresis n=11  2)Wireless iontophoresis n=10  3)Sham iontophoresis n=10 | 2 weeks | 0, 1w, 2w | VAS | - |
| Rio et al. (2017) | N=29 (23yr) | 35.8m (1-120m) | 1)Isometric exercise n=13  2)Isotonic exercise n=16 | 4 weeks | 0, daily pre- and post- exercise, 4w | VAS | VISA-P |
| Rio et al. (2015) | N=6 (27yr) | Not stated | 1)Isometric exercise n=6  2)Isotonic exercise n=6  Same group performed both interventions (cross-over) | Single session of each intervention | 0, post-intervention | VAS | - |
| Rodas et al. (2021) | N=20 (34y) | 23.6m  (>4m) | 1) Bone marrow mesenchymal stem cells + exercise n=10  2) LP-PRP + exercise n=10  The exercises included isotonic, followed by concentric and then eccentric loading | Group 1 received a sham saline injection at day 0 and bone marrow mesenchymal stem cells injection 23 days later  Group 2 received LP-PRP injections at days 0 and 23 | 0, 6m | VAS | VISA-P |
| Scott et al. (2019) | N=18 (32yr) | 2.1y (>6m) | 1)LR-PRP n=20  2)LP-PRP n=21  3)Saline n=20 | Single injection followed by 6 weeks of supervised exercise | 0, 6w, 12w, 6m, 12m | VAS | VISA-P |
| Stasinopoulos & Stasinopoulos (2004) | N=30 (28yr) | Not stated | 1)Eccentric exercise + stretching n=10  2)Pulsed ultrasound n=10  3)Transverse friction | 4 weeks | 0, 4w, 8w, 16w | VAS | - |
| Steunebrink et al. (2013) | N=33 (32.9yr) | 48w | 1)Topical GTN + eccentric exercise n=16  2)Topical Placebo + eccentric exercise n=17 | One patch daily for 12 weeks  Eccentric exercise for ?12 weeks | 0, 6w, 12w, 24w | VAS | VISA-P |
| Thijs et al. (2017) | N=52 (27.3yr) | 23m (3-120m) | 1)f-ESWT + eccentric exercise n=22  2)Sham ESWT + eccentric exercise n=30  Eccentric exercise (decline squat) twice daily for 12 weeks | Exercise (decline squat) for 12 weeks  ESWT 3 sessions over 2 weeks | 0, 6w, 12w, 24w | VAS | VISA-P |
| Van Ark et al. (2016) | N=29 (23yr) | 35.8m (1-120m) | 1)Isometric exercise n=13  2)Isotonic exercise n=16 | 4 weeks | 0, 4w | VAS | VISA-P |
| Van der Worp et al. (2014) | N=43 (31.1yr) | 35.5m (>3m) | 1) f-ESWT + eccentric exercise n=21  2) r-ESWT + eccentric exercise n=22 | 3 sessions over 2 weeks | 0, 7w, 14w | VAS | VISA-P |
| Vetrano et al. (2013) | N=46 (26.9yr) | 18.2m (>3m) | 1)LR-PRP n=23  2)f-ESWT n=23  Both groups received a home exercise programme | PRP 2 injections over 2 weeks  ESWT 3 sessions over 1 week | 0, 2m, 6m, 12m | VAS | VISA-P |
| Visnes et al (2005) | N=29 volleyball athletes (26.6yr) | 73.6m | 1)Eccentric exercise (decline squat) n=13  2)Standard training n=16 | Exercise twice daily for 12 weeks | 0, 1-12w, 18w, 40w | VAS | VISA-P |
| Wang et al. (2007) | N=54 tendons (50 patients) (29.8yr) | 13.8m (6-64m) | 1)ESWT  2)“Conservative treatments” (NSAIDs, exercise, strap, physiotherapy) | Single session of ESWT  Duration of “conservative treatments” not stated | 0, 1m, 3m, 6m, 12m, then once a year up to 53m | VAS | VISA-P |
| Warden et al. (2008) | N=37 (27yr) | 3.8y (>6m) | 1)US + eccentric exercise n=17  2)Sham US + eccentric exercise n=20 | US and exercise for 12 weeks | 0, 12w | VAS | VISA-P |
| Young et al. (2005) | N=17 | Not stated | 1)Eccentric exercise (decline squat) n=9  2)Eccentric exercise (step squat) n=8 | 12 weeks | 0, 12w, 12m | VAS | VISA-P |
| Zwerver et al. (2011) | N=62 (25yr) | 7.7m | 1)f-ESWT n=31  2) Sham ESWT n=31 | ESWT 3 sessions over 2 weeks | 0, 1w, 12w, 22w | VAS | VISA-P |

**Suppl. Table S3. Characteristics of the included randomised controlled trials (participants, interventions, comparators and outcome measures).** *ESWT, extracorporeal shock-wave therapy; months; HA, hyaluronic acid; LP-PRP, leucocyte-poor PRP; LR-PRP, leucocyte-rich PRP; NRS, numerical rating scale; NSAIDs, non-steroidal anti-inflammatory drugs; PRP, platelet-rich plasma; r-ESWT, radial ESWT; US, ultrasound; VAS, visual analogue scale; VISA-P, Victorian Institute of Sports Assessment – Patellar; w, weeks; y, years.*

| Supplementary Table S4. | | | | | | | |  | Achilles Tendinopathy |
| --- | --- | --- | --- | --- | --- | --- | --- | --- | --- |
| Study (year) | Internal Validity  (Cochrane’s Collaboration Tool for Assessing Risk of Bias) | | | | | | | Overall **risk** | Justification |
|  | *Selection*  *bias* | | *Performance*  *bias* | *Detection*  *bias* | *Attrition*  *bias* | *Reporting*  *bias* | *Other* |  |  |
|  | *Random sequence generation* | *Allocation concealment* | *Blinding of patients and staff* | *Blinding of outcome measures* | *Completeness of outcome data* | *Selective reporting* |  |  |  |
| Balius et al  (2016) | High | High | ? | High | Low | Low | Low | **High** | **Random sequence did not produce comparable groups. Single blinded,**  **Low study number.**  **Inclusion of two groups reactive and degenerative.** |
| Bell et al (2013) | Low | Low | Low | Low | Low | Low | Low | **Low** | **-** |
| Beyer et al (2015) | High | High | High | ? | Low | Low | Low | **High** | **Single blinding.**  **Significant difference between baseline outcome score.** |
| Boesen et al (2017) | Low | Low | Low | Low | Low | Low | ? | **Low** | **-** |
| Boesen et al (2019) | Low | Low | Low | Low | Low | Low | ? | **Low** | **-** |
| Bradford et al. (2021) | Low | Low | Low | Low | Low | Low | High | **High** | **Very small population** |
| De Jonge et al (2011) | Low | Low | Low | Low | High | Low | Low | **Low** | **-** |
| De Jonge et al. (2008) | ? | High | High | High | Low | Low | Low | **High** | **Allocation process not described.**  **Blinding not described.** |
| De Vos et al.  (2007) | low | High | High | ? | Low | Low | Low | **High** | **Single blinding, no power calculation.**  **Concealment of outcome measure not described.** |
| Horstmann et al.  (2013) | Low | High | High | High | Low | High | Low | **High** | **No power calculation.**  **Small group size**  **Included both mid portion and insertional tendinopathy.** |
| Herrington et al  (2007) | Low | ? | ? | ? | Low | Low | Low | **High** | **Poor description of allocation and blinding, no power calculation.** |
| Knobloch et al. (2007) | Low | ? | High | ? | Low | Low | Low | **High** | **Non-blinded patients, probably non-blinded outcome assessors** |
| Knobloch et al (2008) | Low | ? | High | Low | Low | High | Low | **High** | **Single blinding.**  **Limited description of concealment process.** |
| Krogh et al (2016) | Low | ? | High | ? | High | Low | Low | **High** | **Single blinded, Variation in baseline VISA-A and VAS score may represent inconsistent allocation. Significant loss of follow up after 6 months.** |
| Mafi et al (2001) | Low | Low | ? | ? | ? | High | Low | **High** | **No power calculation,**  **Results not reported in standard format amendable to interpretation.** |
| Munteanu et al (2015) | Low | Low | Low | Low | Low | Low | Low | **Low** | **-** |
| Petersen et al (2007) | ? | ? | ? | Low | High | Low | Low | **High** | **Randomisation process not clearly defined.**  **Variation in VAS and AOFAS baseline score.** |
| Pearson et al  (2012) | Low | High | High | ? | High | Low | Low | **High** | **Attrition rate 30%.**  **Small study group.**  **Non blinded.** |
| Rabusin et al. (2021) | Low | Low | High | High | Low | Low | Low | **High** | **No blinding of patients, staff or outcome measures** |
| Rompe et al (2007) | Low | Low | ? | ? | Low | Low | Low | **Low** | **-** |
| Rompe et al  (2008) | Low | Low | High | Low | Low | Low | Low | **Low** | **-** |
| Roos et al (2003) | *Low* | *?* | *High* | *?* | *High* | Low | Low | **High** | **Blinding process and concealment not described; study number is lower than the recommended power calculation.** |
| Silbernagel et al (2007) | Low | Low | Low | High | ? | Low | High | **High** | **Single blinding. Power calculation recommendation greater than study cohort.** |
| Silbernagel et al (2001) | ? | High | ? | ? | Low | High | Low | **High** | **Blinding concealment process not adequately described. No power/sample calculation.**  **Significant difference between baseline demographic/outcome measure.** |
| Stevens et al  (2014) | ? | Low | High | Low | Low | Low | Low | **High** | **Sample size less than recommended from power calculation. No description of randomisation.** |
| Tumilty et al  (2016) | Low | ? | Low | Low | High | Low | Low | **High** | **Small groups, rational for 2 exercise groups not explained.** |
| Tumilty et al (2012) | Low | ? | Low | Low | High | Low | Low | **High** | **Small study groups, no power calculation.** |
| Van der Vlist et al (2020a) | Low | Low | Low | Low | Low | Low | Low | **Low** | **-** |
| Van der Vlist et al (2020b) | High |  | High | High | Low | Low | Low | **High** | **High-risk randomisation, non-blinded** |
| Yelland et al  (2009) | Low | Low | Low | ? | Low | Low | Low | **Low** | **-** |
| Zhang et al (2013) | Low | ? | High | ? | Low | Low | Low | **High** | **Single blinding, outcome concealment not extensively described.** |

**Suppl. Table S4. Risk of bias assessment for studies assessing interventions in Achilles tendinopathy**

| Supplementary Table S5. GTPS | | | | | | | |  | GTPS |
| --- | --- | --- | --- | --- | --- | --- | --- | --- | --- |
| Study (year) | Internal Validity  (Cochrane’s Collaboration Tool for Assessing Risk of Bias) | | | | | | | Overall **risk** | Justification |
|  | *Selection*  *bias* | | *Performance*  *bias* | *Detection*  *bias* | *Attrition*  *bias* | *Reporting*  *bias* | *Other* |  |  |
|  | *Random sequence generation* | *Allocation concealment* | *Blinding of patients and staff* | *Blinding of outcome measures* | *Completeness of outcome data* | *Selective reporting* |  |  |  |
| Clifford et al (2019) | Low | Low | ? | ? | High | Low | Low | **High** | **Patient allocation and staff not described.**  **Attrition rate 75%. Small study number.** |
| Ganderton et al (2018) | Low | Low | Low | High | Low | Low | Low | **High** | **Single blinding required due to nature of intervention.**  **Small study number.** |
| Heaver et al. (2021) | Low | Low | High | Low | Low | Low | Low | **High** | **Patients non-blinded** |
| Mellor et al (2018) | Low | High | Low | Low | Low | Low | Low | **Low** | **-** |
| Ramon et al. (2020) | Low | Low | Low | Low | Low | Low | Low | **Low** | **-** |
| Rompe et al (2009) | Low | Low | Low | ? | Low | Low | Low | **Low** | **-** |

**Suppl. Table S5. Risk of bias assessment for studies assessing interventions in GTPS**

| Supplementary Table S6. Patellar Tendinopathy | | | | | | | | | |
| --- | --- | --- | --- | --- | --- | --- | --- | --- | --- |
| Study (year) | Internal Validity  (Cochrane’s Collaboration Tool for Assessing Risk of Bias) | | | | | | | Overall **risk** | Justification |
|  | *Selection*  *bias* | | *Performance*  *bias* | *Detection*  *bias* | *Attrition*  *bias* | *Reporting*  *bias* | *Other* |  |  |
|  | *Random sequence generation* | *Allocation concealment* | *Blinding of patients and staff* | *Blinding of outcome measures* | *Completeness of outcome data* | *Selective reporting* |  |  |  |
| Abas et al. (2016) | Low | ? | High | ? | Low | High | High (no power calculation) | **High** | **Single-blinded, inappropriate reporting of results, concealment process and blinding of assessment not described** |
| Agergaard et al. (2021) | Low | ? | Low | High | Low | Low | Low | **Low** | **-** |
| Bahr et al. (2006) | Low | Low | High | High | Low | Low | Low | **High** | **Non-blinded** |
| Cannell et al (2001) | Low | Low | High | Low | Low | Low | High (no power calculation, small population) | **High** | **Single-blinded, small population** |
| De Vries et al. (2016) | High | ? | High | ? | High | Low | Low | **High** | **Inappropriate randomisation. Single-blinded, significant loss to follow up (29%)** |
| Dragoo et al. (2014) | Low | Low | Low | Low | Low | Low | High (baseline age difference) | **Low** | **-** |
| Frohm et al (2007) | ? | ? | High | ? | Low | Low | High (no power calculation, small population) | **High** | **Single-blinded, small population, randomisation/concealment process and blinding of assessment not described** |
| Holden et al. (2020) | Low | Low | Low | High | Low | Low | Low | **Low** | **-** |
| Jonsson et al (2005) | ? | ? | High | ? | High | Low | High (population not enough for power) | **High** | **Single-blinded, small population, randomisation/concealment process and blinding of assessment not described significant loss to follow up (21%)** |
| Kaux et al. (2015) | ? | ? | ? | ? | ? | Low | High (no power calculation, small population, baseline difference in pain) | **High** | **Small population, baseline differences between groups (may reflect inappropriate randomisation), no details available for selection, performance, detection and attrition bias** |
| Kongsgaard et al. (2009) | Low | ? | High | Low | Low | Low | Low | **Low** | **-** |
| Lee et al. (2017) | ? | ? | High | ? | Low | Low | Low | **High** | **Single-blinded, randomisation/concealment process and blinding of assessment not described** |
| Lopez-Royo et al. (2021) | Low | Low | Low | Low | Low | Low | Low | **Low** | **-** |
| Resteghini et al. (2016) | Low | Low | Low | Low | ? | Low | Low | **Low** | **-** |
| Rigby et al. (2015) | Low | Low | ? | High | ? | Low | High (baseline difference in pain, small population, no power calculation, acute and chronic tendinopathy patients) | **High** | **Single-blinded, baseline difference between groups (may reflect inappropriate randomisation), no details about completeness of outcome data, no power calculation, mixture of acute and chronic tendinopathy patients** |
| Rio et al. (2017) | Low | Low | High | High | High | Low | High (no exclusion criteria) | **High** | **Non-blinded, significant loss to follow up (38%), no exclusion criteria** |
| Rio et al. (2015) | Low | Low | High | High | Low | Low | High (acute and chronic tendinopathy patients) | **High** | **Single-blinded, non-blinded, mixture of acute and chronic tendinopathy patients** |
| Rodas et al. (2021) | Low | ? | High | Low | Low | Low | High (small population) | **High** | **Small population, concealment process not described** |
| Scott et al. (2019) | High | Low | Low | High | Low | High | Low | **High** | **Single-blinded, inappropriate randomisation, inadequate reporting of results (no p values)** |
| Stasinopoulos & Stasinopoulos (2004) | Low | ? | High | Low | Low | High | High (no power calculation, small population, no baseline pain data) | **High** | **Single-blinded, small population, no baseline pain data, non-clinically relevant outcome measures (categorical pain scale)** |
| Steunebrink et al. (2013) | Low | Low | ? | ? | Low | Low | High (baseline difference in pain) | **High** | **Baseline difference between groups (may reflect inappropriate randomisation), blinding processes not described** |
| Thijs et al. (2017) | Low | Low | Low | Low | Low | Low | Low | **Low** | **-** |
| Van Ark et al. (2016) | Low | Low | High | High | High | Low | High (acute and chronic tendinopathy patients) | **High** | **Non-blinded, significant loss to follow up (38%), no exclusion criteria** |
| Van der Worp et al. (2014) | Low | Low | Low | Low | Low | Low | High (population not enough for power) | **Low** | **-** |
| Vetrano et al. (2013) | Low | ? | High | Low | Low | Low | ? (no power calculation but large population) | **Low** | **-** |
| Visnes et al (2005) | High | ? | High | ? | Low | Low | High (no power calculation, small population) | **High** | **Inappropriate randomisation/concealment, single-blinded, blinding of assessment not described small population** |
| Wang et al. (2007) | High | ? | High | ? | Low | High | Low | **High** | **Single-blinded, inappropriate randomisation, inappropriate reporting of results, concealment process and blinding of assessment not described** |
| Warden et al. (2008) | Low | Low | Low | Low | High | Low | High (no power calculation, small sample) | **Low** | **-** |
| Willberg et al. (2011) | Low | Low | High | High | Low | Low | Low | **High** | **Non-blinded** |
| Young et al. (2005) | Low | Low | High | Low | Low | Low | High (no power calculation, small population) | **High** | **Single-blinded, small population** |
| Zwerver et al. (2011) | Low | Low | Low | Low | Low | Low | High (population not enough for power but large) | **Low** | **-** |

**Suppl. Table S6. Risk of bias assessment for studies assessing interventions in patellar tendinopathy**

| Supplementary Table S7. | | | | | | | | | | |
| --- | --- | --- | --- | --- | --- | --- | --- | --- | --- | --- |
| Comparison | Tendinopathy | Outcome measure | Follow up | Number of studies | Overall risk of bias | Inconsistency | Indirectness | Imprecision | Other | Strength of Evidence |
| PRP + Eccentric exercise vs Eccentric exercise + placebo | **Achilles** | **VISA-A** | ST | 3 | High | Low | High | Low | Low | **Low** |
|  |  | **VISA-A** | MT | 3 | High | Low | High | Low | Low | **Low** |
| Low level laser therapy + eccentric exercise vs Eccentric exercise + sham | **Achilles** | **VISA-A** | ST | 2 | High | High  (-2) | Low | Low | Low | **V. Low** |
| Corticosteroid injection vs exercise | **GTPS** | **VAS** | ST | 2 | Low | High  (-2) | Low | Low | Low | **Low** |
|  |  | **VAS** | LT | 2 | Low | High  (-2) | Low | Low | Low | **Low** |
| Shock wave therapy + eccentric exercise vs eccentric exercise | **Patellar** | **VAS** | ST | 2 | High | Low | Low | Low | Low | **Mod** |
|  |  | **VISA-P** | ST | 2 | High | Low | Low | Low | Low | **Mod** |
| Isotonic exercise  Vs Isometric exercise | **Patellar** | **VAS** | ST | 3 | High | High | Low | Low | Low | **Low** |

**Suppl. Table S7. Results of assessing the certainty (strength) of evidence according to the GRADE tool for each comparison of interventions**

| Achilles Tendinopathy  **Supplementary table S8** |  |  | | |  | | |
| --- | --- | --- | --- | --- | --- | --- | --- |
| Comparison  Intervention vs control. | Study | Pain (VAS 0-10) | | | Function (VISA-A 0-100) | | |
|  | Follow up | ST | MT | LT | ST | MT | LT |
| Heel lifts (1) vs ECC (2) | Rabusin et al. (2021) | ↓ | - | - | ↑ | - | - |
| ABI + ECC (1) vs ECC + placebo (needling) (2) | Bell et al. (2013)(44) | - | - | - | - | - | - |
| ECC (1) vs ECC + MCVC (2) | Balius et al  (2016) | ↓ | ↔ | - | ↓ | ↔ | - |
| ECC (1) vs Passive stretching + MCVC. | Balius et al  (2016) | ↔ | ↔ | - | ↔ | ↔ | - |
| ECC + MCVC (1) vs Passive stretching + MCVC (2). | Balius et al  (2016) | ↔ | ↑ | - | ↔ | ↔ | - |
| ECC (1) vs ECC + MCVC (2) | Balius et al  (2016) | ↔ | ↔ | - | ↔ | ↔ | - |
| ECC (1) vs Passive stretching + MCVC. | Balius et al  (2016) | ↔ | ↔ | - | ↔ | ↔ | - |
| ECC + MCVC (1) vs Passive stretching + MCVC (2) | Balius et al  (2016) | ↔ | ↔ | - | ↔ | ↔ | - |
| CSI + ECC (1) vs PRP + ECC (2) | Boesen et al (2017) | ↔ | ↔ | - | ↑ | ↔ | - |
| HVI + ECC (1) VS HVI + ECC (2) | Boesen et al (2017) | ↓ | ↓ | - | ↑ | ↑ | - |
| HVI + ECC (1) VS PRP +ECC (2) | Boesen et al (2017) | ↑ | ↑ | - | ↓ | ↓ | - |
| HVI (steroid) + ECC (1) VS HVI (no steroid) +ECC (2) | Boesen et al (2019) | ↓ |  | - |  |  |  |
| ECC (1) vs Resistance training (heavy slow) (2) | Beyer et al (2015) | - | ↑ | ↑ | - | ↓ | ↓ |
| ECC (1) vs ECC + Splint (2) | De Jonge et al (2008) | - | - | - | ↔ | ↔ | - |
| PRP + ECC (1) vs saline-I + ECC (2) | De Jonge et al (2011) | - | - | - | ↔ | ↔ | ↔ |
| ECC (1) vs Night splint + ECC (2). | De Vos (2007) | - | - | - | ↔ | - | - |
| ECC (1) vs Vibration | Horstmann et al (2013) | ↓ | - | - | - | - | - |
| Vibration (1) vs Wait and see (2) | Horstmann et al (2013) | ↑ | - | - | - | - | - |
| ECC (1) vs Wait + See (2) | Horstmann et al (2013) | ↓ | - | - | - | - | - |
| ECC (1) vs (2) Deep frictional massage -control | Herrington et al (2007) | - | - | - | ↑ | - | - |
| ECC (1) vs Air heel brace + ECC (2 ) | Knobloch et al (2008) | ↔ | - | - | - | - | - |
| PRP (1) + ECC vs (2) saline + ECC | Krogh et al (2016) | ↔ | - | - | ↔ | ↔ | ↔ |
| ECC (1) vs Physical therapy (stretches) (2) | Kedia et al (2014) | - | - | - | - | - | - |
| ECC (1) vs Concentric training | Mafi et al (2001) | - | - | - | - | - | - |
| Customised foot (1) + ECC vs orthoses Sham + ECC | Munteanu et al (2015) | - | - | - | ↔ | ↔ | ↔ |
| ECC (1) vs Airheel brace | Petersen et al (2007) | - | ↔ | ↔ | - | - | - |
| ECC (1) vs ECC + air heel brace | Petersen et al (2007) | - | ↔ | ↔ | - | - | - |
| Airheel brace (1) vs ECC + Air heel brace | Petersen et al (2007) | - | ↔ | ↔ | - | - | - |
| PRP+ ECC (1) vs ECC | Pearson et al  (2012) | - | - | - | ↔ | - | - |
| ECC (1) vs ESWT (2) | Rompe et al (2007) | - | ↔ | - | - | ↔ | - |
| ESWT (1) vs Wait + see (2) | Rompe et al (2007) | - | ↓ | - | - | ↑ | - |
| ECC (1) vs ESWT (2) | Rompe et al (2007) | - | ↓ | - | - | ↑ | - |
| ECC (1) vs ESWT + ECC(2) | Rompe et al  (2009) | - | ↑ | - | - | ↓ | - |
| ECC (1) vs ECC + splint (2) | Roos et al (2003) | - | - | - | - | - | - |
| ECC (1) vs Splint | Roos et al (2003) | - | - | - | - | - | - |
| ECC + Splint (1) vs Splint | Roos et al (2003) | - | - | - | - | - | - |
| ECC/Con (1) vs Active rest (2) | Silbernagel et al (2007) | ↔ | ↔ | ↔ | ↔ | ↔ | ↔ |
| Exercise group (1) (overload) vs ECC (2) | Silberngael et al (2001) | - | ↔ | ↓ | - | - | - |
| ECC vs ECC (1) + Exercise tolerance (2) | Stevens et al  (2014) | ↔ | - | - | ↔ | - | - |
| Placebo + ECC2 (1) LIP + ECC1 (2) | Tumilty et al  (2016) | ↔ | ↔ | - | ↔ | ↑ | - |
| Placebo + ECC2 (1) vs LIP + ECC2 (2) | Tumilty et al  (2016) | ↔ | ↓ | - | ↔ | ↑ | - |
| Placebo + ECC2 (1)  Placebo + ECC1 (2) | Tumilty et al  (2016) | ↔ | ↔ | - | ↔ | ↑ | - |
| Placebo + ECC1 (1) vs LIP + ECC1 (2) | Tumilty et al  (2016) | ↔ | ↔ | - | ↔ | ↔ | - |
| Placebo + ECC1 (1)  vs LIP + ECC2 (2) | Tumilty et al  (2016) | ↔ | ↔ | - | ↔ | ↔ | - |
| LIP + ECC2 (1) vs LIP + ECC1 (2) | Tumilty et al  (2016) | ↔ | ↔ | - | ↔ | ↔ | - |
| LL-TT + ECC (1) vs placebo + ECC (2) | Tumilty et al (2012) | ↔ | ↔ | ↔ | ↔ | ↔ | ↔ |
| High volume injection (1) + ECC vs Placebo injection + ECC (2) | Van der Vlist (2020a) | - | - | - | ↔ | ↔ | - |
| ECC + Prolotherapy injections (1) vs ECC (2) | Yelland et al  (2009) | - | - | - | ↔ | ↔ | ↑ |
| ECC (1) vs Acupuncture + ECC (2) | Zhang et al (2013) | ↑ | - | - | ↔ | ↔ | - |
| Isometric exercise (knee flexed) (1) vs isometric exercise (knee extended) (2) | Bradford et al. (2021) | ↔ | - | - | - | - | - |
| Isometric exercise (ankle dorsiflexed) (1) vs isometric exercise (tiptoes) (2) | Van der Vlist (2020b) | ↔ | - | - | - | - | - |
| Isometric exercise (ankle dorsiflexed) (1) vs isotonic exercise (2) | Van der Vlist (2020b) | ↔ | - | - | - | - | - |
| Isometric exercise (ankle dorsiflexed) (1) vs rest (2) | Van der Vlist (2020b) | ↔ | - | - | - | - | - |
| Isotonic exercise vs isometric exercise (tiptoes) (2) | Van der Vlist (2020b) | ↔ | - | - | - | - | - |
| Rest vs isometric exercise (tiptoes) (2) | Van der Vlist (2020b) | ↔ | - | - | - | - | - |
| Isotonic exercise (1) vs rest (2) | Van der Vlist (2020b) | ↔ | - | - | - | - | - |

**Suppl. Table S8. Results of compared interventions from included studies in Achilles tendinopathy shown qualitatively based on direction of effect [“Pain (VAS 0-10)” and “Function (VISA-P 0-100)”].** For pain VAS the “down” arrow (↓) favours intervention “1” and the “up” arrow (↑) intervention “2” at statistical significance; for VISA-P, the “up” arrow (↑) favours intervention “1” and the “down” (↓) arrow intervention “2” at statistical significance. The “↔” arrow denotes no statistical significance.

*BM-MSC, bone marrow mesenchymal stem cells; ESWT, extracorporeal shock-wave therapy; LL-LT. Low laser therapy; m, months; NRS, numerical rating scale; NSAIDs, non-steroidal anti-inflammatory drugs; PNE, percutaneous needle electrolysis; PRP, platelet-rich plasma; US, ultrasound; USGT, VAS, visual analogue scale; VISA-P, Victorian Institute of Sports Assessment – Patellar; w, weeks; y, years*

| GTPS  **Supplementary Table S9** |  |  | | |  | | |
| --- | --- | --- | --- | --- | --- | --- | --- |
| Comparison  Intervention vs control. | Study | Pain (VAS 0-10) | | | Function (VISA-G 0-100) | | |
|  | Follow up | ST | MT | LT | ST | MT | LT |
| EDX (1) vs W&S (2) | Mellor et al (2018) | ↓ | ↓ | ↓ | ↑ | ↑ | ↔ |
| CSI (1) vs W&S (2) | Mellor et al (2018) | ↓ | ↔ | ↓ | ↑ | ↑ | ↔ |
| EDX (1) vs CSI (1) | Mellor et al (2018) | ↔ | ↔ | ↔ | ↔ | ↑ | ↔ |
| Home training (1) vs CSI (2) | Rompe et al (2009) | ↑ | ↔ | ↓ | - | - | - |
| CSI (1) vs r-ESWT (2) | Rompe et al (2009) | ↓ | ↑ | ↑ | - | - | - |
| Home training (1) vs ESWT | Rompe et al (2009) | ↔ | ↑ | ↔ | - | - | - |
| Exercise Group (1) vs Sham Group (2) | Ganderton et al (2018) | - | - | - | ↔ | ↑ | - |
| Isometric exercise programme (1) vs  Isotonic exercise programme (2) | Clifford et al (2019) | - | - | - | ↔ | ↔ | - |
| f-ESWT + exercise (1) vs sham ESWT + exercise | Ramon et al. (2020) | ↓ | - | - | - | - | - |
| f-ESWT + exercise (1) vs CSI +exercise | Heaver et al. (2021) | ↔ | - | ↓ | - | - | - |

**Suppl. Table S9. Results of compared interventions from included studies in GTPS shown qualitatively based on direction of effect [“Pain (VAS 0-10)” and “Function (VISA-P 0-100)”].** For pain the “down” arrow (↓) favours intervention “1” at statistical significance, the “up” arrow (↑) intervention “2” and the “equal” arrow (↔) shows no statistical difference for the compared interventions; for VISA-G, the “up” arrow (↑) favours intervention “1” and the “down” (↓) arrow intervention “2”. The “↔” arrow denotes no statistical significance.

*ESWT, extracorporeal shock-wave therapy; EDX, education, m, months; LP-PRP, NRS, numerical rating scale; NSAIDs, non-steroidal anti-inflammatory drugs; US, ultrasound; VAS, visual analogue scale; VISA-P, Victorian Institute of Sports Assessment – Patellar; w, weeks; y, years.*

| Patellar tendinopathy  **Supplementary Table S10** |  |  | | |  | | |
| --- | --- | --- | --- | --- | --- | --- | --- |
| Comparison | Study | Pain (VAS 0-10) | | | Function (VISA-P 0-100) | | |
|  | Follow up | ST | MT | LT | ST | MT | LT |
| Eccentric (1) vs Concentric (2) exercise | Cannell et al (2001) | ↔ | - | - | - | - | - |
|  | Jonsson et al (2005) | ↓ | - | - | ↑ | - | - |
| f-ESWT + Eccentric exercise (1) vs sham ESWT + Eccentric exercise (2) | Lee et al. (2017) | ↔ | - | - | ↔ | - | - |
|  | Thijs et al. (2017) | ↔ | ↔ | - | ↔ | ↔ | - |
| Isometric (1) vs Isotonic (2) exercise – immediate post-intervention outcomes | Holden et al. (2020) | ↔ | - | - | - | - | - |
|  | Rio et al. (2015) | ↓ | - | - | - | - | - |
|  | Rio et al. (2017) | ↓ | - | - | ↔ | - | - |
| Isometric (1) vs Isotonic (2) exercise – 4-week outcomes | Van Ark et al. (2016) | ↔ | - | - | ↔ | - | - |
| Eccentric exercise decline squat (1) vs standard/step squat (2) | Young et al. (2005) | ↑ | - | ↔ | ↔ | - | ↑ |
| Eccentric exercise (1) vs Surgery (2) | Bahr et al. (2006) | - | - | ↔ | ↔ | ↔ | - |
| PTLE Progressive tendon loading exercises (1) vs Eccentric exercise therapy (2) | Breda et al.  (2020) | - | - | - | - | - | - |
| BM-MSC + exercise (1) vs LP-PRP injection + exercise (2) | Rodas et al. (2021) | - | ↔ | - | - | ↔ | - |
| LR-PRP + exercise (1) vs Saline + exercise (2) injection | Scott et al. (2019) | ↔ | ↔ | ↔ | ↔ | ↔ | ↔ |
| LP-PRP + exercise (1) vs Saline + exercise (2) injection | Scott et al. (2019) | ↔ | ↔ | ↔ | ↔ | ↔ | ↔ |
| LR-PRP + exercise (1) vs LP-PRP + exercise (2) | Scott et al. (2019) | ↔ | ↔ | ↔ | ↔ | ↔ | ↔ |
| Patellar strap (1) vs Sports taping (2) | De Vries et al. (2016) | ↔ | - | - | - | - | - |
| Patellar strap (1) vs no treatment (2) | De Vries et al. (2016) | ↔ | - | - | - | - | - |
| Sports taping (1) vs no treatment (2) | De Vries et al. (2016) | ↔ | - | - | - | - | - |
| Electrophysiotherapy + eccentric exercise (1) vs USGT + eccentric exercise (2) | Abat et al. (2016) | - | - | - | ↓ | - | - |
| Dry Needling + Eccentric exercise (1) vs PRP + Eccentric exercise (2) | Dragoo et al. (2014) | ↔ | ↔ | - | ↓ | ↔ | - |
| Eccentric exercise (1) vs Eccentric device (overload) (2) | Frohm et al (2007) | ↔ | - | - | ↔ | - | - |
| Single PRP injection + Eccentric exercise (1) vs two PRP injections + Eccentric exercise (2) | Kaux et al. (2015) | ↓ | - | ↓ | ↔ | - | ↔ |
| Corticosteroid injection (1) vs Eccentric exercise (2) | Kongsgaard et al. (2009) | - | ↔ | - | - | ↓ | - |
| Corticosteroid injection (1) vs Heavy Slow Resistance exercise (2) | Kongsgaard et al. (2009) | - | ↑ |  | - | ↓ | - |
| Heavy Slow Resistance (1) vs Eccentric (2) exercise | Kongsgaard et al. (2009) | ↔ | ↔ | - | - | ↔ | - |
| Autologous blood + eccentric (1) vs Saline + eccentric (2) | Resteghini et al. (2016) | ↔ | - | ↔ | ↔ | - | ↔ |
| Wired (1) vs Wireless (2) iontophoresis | Rigby et al. (2015) | ↔ | - | - | - | - | - |
| Wired (1) vs Sham (2) iontophoresis | Rigby et al. (2015) | ↔ | - | - | - | - | - |
| Wireless (1) vs Sham (2) iontophoresis | Rigby et al. (2015) | ↔ | - | - | - | - | - |
| Eccentric exercise + stretching (1) vs pulsed US (2) | Stasinopoulos & Stasinopoulos (2004) | ↓ | - | - | - | - | - |
| Eccentric exercise + stretching (1) vs transverse friction (2) | Stasinopoulos & Stasinopoulos (2004) | ↓ | - | - | - | - | - |
| Pulsed US (1) vs transverse friction (2) | Stasinopoulos & Stasinopoulos (2004) | ↔ | - | - | - | - | - |
| Topical GTN + Eccentric exercise (1) vs Placebo + Eccentric exercise (2) | Steunebrink et al. (2013) | - | ↔ | - | - | ↔ | - |
| f-ESWT + Eccentric exercise (1) vs r-ESWT + Eccentric exercise (2) | Van der Worp et al. (2014) | ↔ | - | - | ↔ | - | - |
| LR-PRP (1) vs ESWT (2) | Vetrano et al. (2013) | ↔ | ↓ | ↓ | ↔ | ↑ | ↑ |
| Eccentric exercise (1) vs Standard training (2) | Visnes et al (2005) | - | - | - | ↔ | - | - |
| ESWT (1) vs “Conservative treatments” (2) | Wang et al. (2007) | - | - | ↓ | - | - | ↑ |
| US + Eccentric exercise (1) vs Sham US + Eccentric Exercise (2) | Warden et al. (2008) | ↔ | - | - | ↔ | - | - |
| Sclerosing polidocanol injections (1) vs Arthroscopic surgery (2) | Willberg et al. (2011) | - | - | ↑ | - | - | - |
| f-ESWT (1) vs Sham ESWT (2) | Zwerver et al. (2011) | ↔ | ↔ | - | ↔ | ↔ | - |

**Suppl. Table S10. Results of compared interventions from included studies in patellar tendinopathy shown qualitatively based on direction of effect [“Pain (VAS 0-10)” and “Function (VISA-P 0-100)”].** For pain the “down” arrow (↓) favours intervention “1” at statistical significance, the “up” arrow (↑) intervention “2” and the “equal” arrow (↔) shows no statistical difference for the compared interventions; for VISA-P, the “up” arrow (↑) favours intervention “1” and the “down” (↓) arrow intervention “2”. The “↔” arrow denotes no statistical significance.

*BM-MSC, bone marrow mesenchymal stem cells; ESWT, extracorporeal shock-wave therapy; f-ESWT, focal ESWT; GTN, glyceryl trinitrate; m, months; LP-PRP, leucocyte-poor PRP; LR-PRP, leucocyte-rich PRP; NRS, numerical rating scale; NSAIDs, non-steroidal anti-inflammatory drugs; PRP, platelet-rich plasma; r-ESWT, radial ESWT; US, ultrasound; USGT, ultrasound-guided galvanic therapy; VAS, visual analogue scale; VISA-P, Victorian Institute of Sports Assessment – Patellar; w, weeks; y, year*
